# Mesenchymal stromal cell secretome-induced synaptogenesis is mediated by thrombospondin-1

**DOI:** 10.1016/j.isci.2025.113401

**Published:** 2025-08-19

**Authors:** Diogo Tomé, Luís F. Martins, Miguel Aroso, Henrique Santos, Rui O. Costa, João L. Afonso, Sofia C. Serra, Paulo Aguiar, Carlos B. Duarte, João Peça, Paulo S. Pinheiro, António J. Salgado, Ramiro D. Almeida

**Affiliations:** 1 iBiMED- Institute of Biomedicine, Department of Medical Sciences, University of Aveiro, Aveiro, Portugal; 2 CNC, Center for Neuroscience and Cell Biology, University of Coimbra, Coimbra, Portugal; 3 CiBB - Centre for Innovative Biomedicine and Biotechnology, University of Coimbra, Coimbra, Portugal; 4 Institute for Interdisciplinary Research, University of Coimbra, Coimbra, Portugal; 5 INEB- Instituto de Engenharia Biomédica, Universidade do Porto, Porto, Portugal; 6 Neuroengineering and Computational Neuroscience Lab, i3S- Instituto de Investigação e Inovação em Saúde, Universidade do Porto, Porto, Portugal; 7 Life and Health Sciences Research Institute (ICVS), School of Medicine, University of Minho, Braga, Portugal; 8 ICVS/3B’s-PT Government Associate Laboratory, Braga/Guimarães, Portugal; 9 Department of Life Sciences, University of Coimbra, Coimbra, Portugal

**Keywords:** Natural sciences, Biological sciences, Neuroscience, Cellular neuroscience

## Abstract

Mesenchymal stromal cells (MSCs) have been proposed as a promising therapeutic tool for traumatic disorders of the nervous system, due to their neuro-regenerative properties. These outcomes have been extensively connected to paracrine mechanisms. Because synaptic reintegration of injured axons into existing circuitry is required for functional recovery, we investigated the synaptogenic potential of MSC secretome. Using neuronal primary cultures, hippocampal organotypic slices, and human cortical brain organoids, we found that MSC secretome induces presynaptic differentiation. Moreover, the number of axodendritic synapses also increases after treatment with MSC secretome. This increase in synapses correlates with an enhancement in synaptic activity, revealing that newly formed synapses are functional. Finally, we unraveled the mechanism underlying this effect and identified thrombospondin-1 (TSP1) as the major synaptogenic factor in MSC secretome. Together, our findings demonstrate that MSC secretome displays synaptogenic properties, pointing to a potential role as a cell-free therapy to restore lost synaptic connectivity.

## Highlights

• MSC secretome induces axonal branching and presynaptic differentiation in CNS neurons
• The synaptogenic effect of the secretome is also observed in human brain organoids
• MSC secretome promotes the formation of functional excitatory synaptic contacts
• TSP1 mediates MSC secretome-induced synaptogenesis through interaction with α2δ-1

## Graphical abstract

**Figure:**
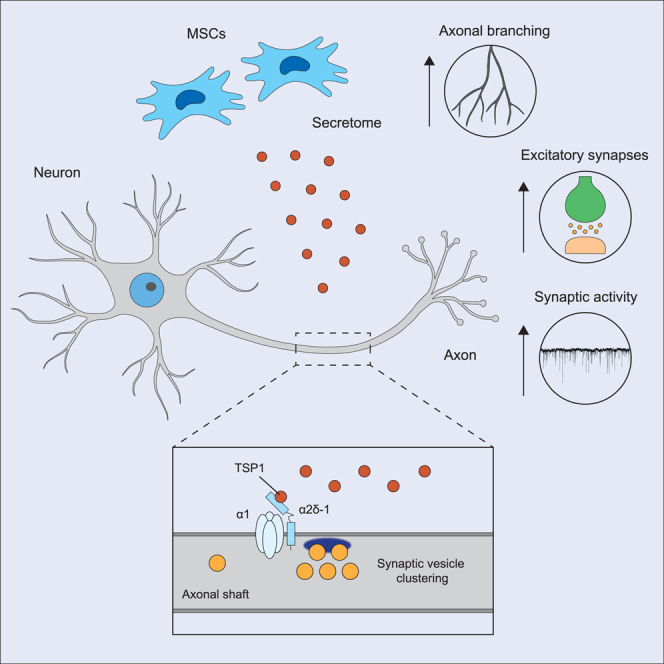

## Introduction

Injury to the central nervous system (CNS), such as spinal cord injury (SCI) or traumatic brain injury, has debilitating and costly effects on human life. Permanent loss of function in these conditions arises from the incapability of damaged axons to regrow and reconnect with their targets. This lack of regeneration has been attributed to the poor intrinsic growth capacity of adult neurons, lack of growth-promoting factors, and the presence of growth inhibitors associated with myelin and glial scarring.^1^^,^^2^^,^^3^ Therefore, the development of a growth-permissive environment for injured axons is critical for achieving functional restoration.

Mesenchymal stromal cells (MSCs) are self-renewing, multipotent precursors present in the stromal fraction of several adult tissues, including bone marrow, dental pulp, adipose tissue, placenta, and umbilical cord.^4^ They have drawn intense interest for the last few years due to their regenerative effects in the nervous system.^5^^,^^6^ A growing number of studies have revealed promising effects of MSC transplantation for the recovery of the injured spinal cord.^7^ However, the mechanism through which these cells exert their therapeutic potential remains unclear. Several reports demonstrated an ability of MSCs to adopt a neuronal or glial phenotype following transplantation into the nervous system.^8^^,^^9^ Whether these MSC-derived neuron-like cells can become integrated into functional synaptic circuits and promote the observed neurological improvements remains to be elucidated. Recently, the regenerative potential of MSCs has been attributed to their paracrine activity.^10^^,^^11^^,^^12^^,^^13^ Several growth and neurotrophic factors, including brain-derived neurotrophic factor (BDNF), nerve growth factor (NGF), neurotrophin-3 (NT-3), glial-cell-line-derived neurotrophic factor (GDNF) and vascular endothelial growth factor (VEGF), cytokines (interleukin (IL)-6, -8, -9, -13, and -27), extracellular matrix proteins and immunomodulatory factors (transforming growth factor β1 (TGF-β1), ciliary neurotrophic factor [CNTF], and interleukin-10 [IL-10]) were identified in MSC secretome.^14^^,^^15^ This wide spectrum of compounds confers MSC secretome neuro-regenerative, anti-apoptotic, immunomodulatory, and angiogenic properties. We have previously shown that the secretome of Wharton-jelly-derived MSCs promotes axonal outgrowth of CNS neurons, through the action of BDNF.^10^ Moreover, exosomes isolated from bone-marrow-derived MSCs also enhance axonal growth of cortical neurons *in vitro,*^16^ demonstrating that the vesicular fraction of MSC secretome can also have regenerative properties. Thus, secretome administration emerges as an alternative approach to MSC grafting that presents several disadvantages, such as the high number of cells required for transplantation and their low survival rate when delivered into a damaged tissue.^17^

The establishment of the neuronal circuitry after injury requires axon growth to be accompanied by synapse formation to achieve functional recovery. In the developing nervous system, a number of molecules involved in synaptogenesis have been identified,^18^ and several reports indicate that the same developmental mechanisms operate in adulthood to induce synaptic reorganization after injury.^19^^,^^20^^,^^21^ However, until now, the synaptogenic potential of MSC secretome is poorly characterized. An important observation came from Mauri and colleagues who, by taking advantage of a co-culture system between rat bone-marrow-derived MSCs and hippocampal neurons, demonstrated that the paracrine action of MSCs enhances GABAergic synaptogenesis and transmission,^22^ suggesting that soluble factors released by MSCs may display synaptogenic properties. In this study, we demonstrated that the secretome of a population of mesenchymal progenitors residing in the Wharton jelly of the umbilical cord,^23^ known as human umbilical cord perivascular cells (HUCPVCs), promotes excitatory synapse formation in developing CNS neurons. This increase in the number of synapses correlated with an enhancement in synaptic activity, demonstrating that the newly formed synapses are functional. We also found that thrombospondin-1 (TSP1), an astrocyte-derived glycoprotein involved in excitatory synaptogenesis,^24^^,^^25^^,^^26^ is a key molecule responsible for the observed effects. Finally, we demonstrate that the secretome possesses the capacity to induce synaptogenesis in human brain organoids, thereby underscoring its potential relevance to human neurological disorders characterized by synaptic loss. Overall, these results show that HUCPVC secretome has synaptogenic properties and may constitute a new approach to restore the ability of injured axons to form functional synapses with target cells.

## Results

### HUCPVC secretome induces axonal branching in CNS neurons

**Figure 1:**
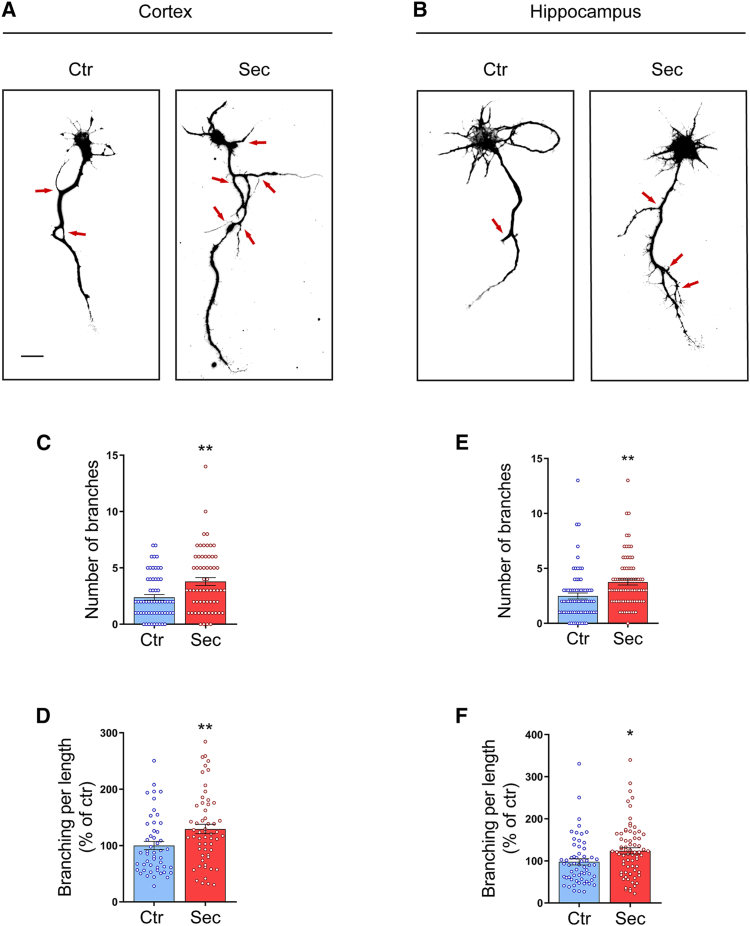
HUCPVC secretome induces axonal branching in CNS neurons (A and B) Effect of HUCPVC secretome in axonal branching of both cortical and hippocampal neurons. At DIV3, neurons were stimulated for 14 h with the secretome. Axons were identified by immunocytochemistry using an antibody against Tau. Images were taken from random neurons using an AxioObserver Z1 fluorescent microscope with a PlanApochromat 20x objective. Scale bars, 25 μm. (C–F) Quantification of axonal branch number (C, E) and branching per length (D, F). Results show that global application of HUCPVC secretome to both cortical and hippocampal neurons for 14 h causes an increase in axonal branching. Axonal branching analysis was performed with ImageJ 1.45e software. Bars represent the mean ± SEM of 50 (Ctr) or 57 (Sec) neurons randomly selected of four independent cultures (C and D) or the mean ± SEM of 59 (Ctr) or 72 (Sec) neurons randomly selected of five independent cultures (E and F); (C) ∗∗ represents *p* = 0.0015; (D) ∗∗ represents *p* = 0.0088; (E) ∗∗ represents *p* = 0.0013, and (F) ∗ represents *p* = 0.0152 by unpaired t test when compared to Ctr.

Proper brain function requires the establishment of a complex neural circuitry. To form this circuitry, a single neuron must communicate with multiple postsynaptic partners, and this is only achieved through extensive branching of its axon.^27^ Therefore, in the context of restoring lost axonal connectivity, we first asked if HUCPVC secretome is able to induce axonal branching of CNS neurons. The HUCPVC used for secretome collection exhibit characteristic properties of MSCs, since they satisfy the minimal criteria defined by the International Society for Cell and Gene Therapy (ISCT) to be classified as such.^28^ They are positive for CD90, CD73, CD105, and CD44 cell surface markers, lack the expression of HLA-DR and CD45 surface markers (Figure S1A), are plastic adherent, and differentiate *in vitro* into adipocytes and osteocytes (Figure S1B). We stimulated rat embryonic cortical (Figure 1A) and hippocampal (Figure 1B) neurons with HUCPVC secretome (Sec) for 14 h. Axons were immunostained against the axonal-specific marker Tau, and branching was assessed by using a quantification protocol developed in our laboratory (described in detail in the STAR Methods section). To exclude any effect caused by media exchange, in the control condition (Ctr) culture medium was also replaced by fresh neurobasal medium. We observed an increase in the number of branches of both cortical (Figure 1C) and hippocampal (Figure 1E) axons treated with HUCPVC secretome, when compared to control axons. Additionally, the number of branches per axon length also increased in axons of cortical (129.3%, *p* = 0.0088) and hippocampal (123.7%, *p* = 0.0152) neurons (Figures 1D and 1F). These observations demonstrate that HUCPVC secretome administration promotes significant axonal branching in developing CNS neurons.

### HUCPVC secretome promotes the formation of presynaptic sites in cultured CNS neurons and hippocampal slices

**Figure 2:**
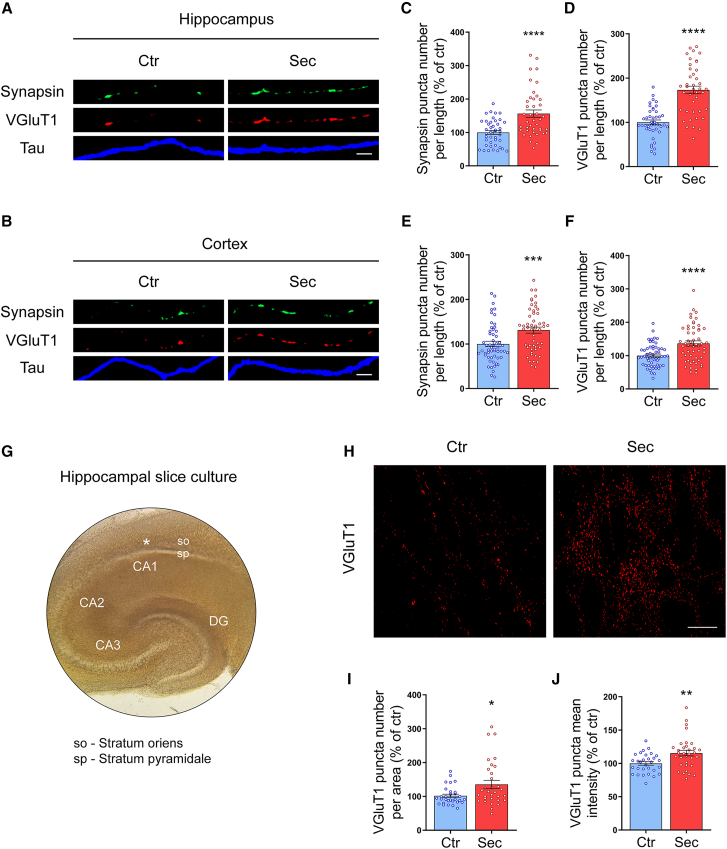
HUCPVC secretome promotes formation of presynaptic sites in CNS neurons (A and B) Effect of HUCPVC secretome in presynaptic assembly. At DIV7, neurons were stimulated for 6 h with the secretome. The formation of presynaptic clusters was assessed by immunocytochemistry using an antibody against the synaptic vesicle markers synapsin (green) or VGluT1 (red), while axons were identified using an antibody against tau (blue). Images were acquired from random axons using an AxioObserver Z1 fluorescent microscope with a PlanApochromat 63x oil objective. Scale bars, 2.5 μm. (C–F) Quantification of synapsin (C and E) and VGluT1 (D and F) puncta number per axonal length. Results show that global application of secretome for 6 h significantly increases the number of synapsin and VGluT1 puncta, demonstrating that secretome stimulation induces the formation of new presynaptic sites in both hippocampal and cortical neurons. Puncta analysis was performed with ImageJ 1.45e software. Results are expressed as % of control. Bars represent the mean ± SEM of 42 images from randomly selected areas of three independent cultures (C and D) or the mean ± SEM of 55 (Ctr) or 53 (Sec) images from randomly selected areas of four independent cultures (E and F); (C) ∗∗∗∗ represents *p* < 0.0001; (D) ∗∗∗∗ represents *p* < 0.0001; (E) ∗∗∗ represents *p* = 0.0008, and (F) ∗∗∗∗ represents *p* < 0.0001 by unpaired t test when compared to Ctr. (G) Representative DIC image of rat hippocampal organotypic slice culture displaying the region where the analysis was performed (asterisk). (H) Effect of HUCPVC secretome on excitatory presynaptic differentiation in the CA1 area. At DIV 4, hippocampal slices were treated with HUCPVC secretome for 48 h. The formation of excitatory presynaptic sites was evaluated by immunocytochemistry using an antibody against the synaptic vesicle marker VGluT1 (red). Representative images were acquired from the stratum oriens of the CA1 area using a Carl Zeiss LSM 710 confocal microscope with a PlanApochromat 63x oil objective. Scale bars, 20 μm. (I and J) Quantitative data of VGluT1 puncta number (I) and mean intensity (J). Results show that treatment of hippocampal slices with the secretome for 48 h significantly increases the number and intensity of VGluT1 puncta in the stratum oriens of the CA1 area, demonstrating that the soluble molecules released by HUCPVCs induce presynaptic differentiation in the hippocampus. Puncta analysis was performed with ICY software. Results are expressed as % of control. Bars represent the mean ± SEM of 30 (Ctr) or 32 (Sec) images (2–3 images per slice) of five independent cultures; (I) ∗ represents *p* = 0.0192 and (J) ∗∗ represents *p* = 0.004 by Mann-Whitney unpaired t test when compared to Ctr.

Synapse formation relies on the presence of appropriate signals, so that a portion of the growing axon can be converted into a presynaptic specialization. In the presence of these stimuli, recruitment of presynaptic active zone proteins and neurotransmitter-containing synaptic vesicles (SVs) occur along the axon, establishing new presynaptic sites.^18^ Thus, we sought to evaluate the effect of HUCPVC secretome on presynaptic assembly in order to characterize its synaptogenic potential. To do this, we plated the neurons in the middle of the coverslip to create a “pseudo-explant” that allows the isolation and visualization of distal axons. In this culture system, the cell bodies and dendrites stay in the middle, while the axons grow toward the periphery of the coverslip, allowing the study of axon-related mechanisms (Figure S2). At DIV7, cortical and hippocampal neurons were stimulated with HUCPVC secretome for 6 h. Fresh neurobasal medium was added in the control conditions. To assess the formation of presynaptic clusters, neurons were immunostained against the SV markers synapsin or vesicular glutamate transporter 1 (VGluT1) (Figures 2A and 2B). Tau immunostaining was used as an axonal marker. Secretome treatment of hippocampal neurons increased the number of synapsin puncta (156.4%, *p* < 0.0001) and VGluT1 puncta (173.1, *p* < 0.0001) per axon length (Figures 2C and 2D), when compared to control axons. This synaptogenic effect of HUCPVC secretome was also observed in cortical neurons, where secretome stimulation resulted in a significant increase in synapsin (131.1%, *p* = 0.0008) and VGluT1 (136.6%, *p* < 0.0001) puncta number per axon length (Figures 2E and 2F). To exclude any non-specific effect caused by the addition of concentrated medium in which HUCPVC secretome was collected (see STAR Methods), hippocampal neurons were treated at DIV7 with two times concentrated neurobasal A medium containing 1% kanamycin (MNB 2x) for 6 h (Figure S3A). This medium was concentrated in the same way as the secretome, but it did not come into touch with cells. We did not observe differences in the number of synapsin (Figure S3B) or VGluT1 (Figure S3C) puncta per axon length after MNB 2x stimulation, when compared to the control condition. Taken together, these data show that HUCPVC secretome induces an increase in SVs clustering along the axonal shaft of both hippocampal and cortical neurons, indicating that it promotes the formation of new presynaptic sites in CNS neurons.

To determine if HUCPVC secretome could also trigger presynaptic differentiation in a model where the *in vivo* neuronal circuitry is maintained, we used hippocampal organotypic slice cultures (Figure 2G). These cultures preserve most of the cellular architecture and functional properties of the corresponding hippocampal circuits *in vivo*, allowing the study of neuronal and glial cells within their native three-dimensional (3D) environment. Furthermore, the development of slice cultures closely resembles the equivalent timeline *in vivo*.^29^ At DIV4, hippocampal slices were treated with HUCPVC secretome or neurobasal medium (Control) for 48 h. The density of excitatory presynaptic terminals formed in the *stratum oriens* of the CA1 layer was assessed by immunostaining the slices against the presynaptic marker VGluT1 (Figure 2H). This region contains the basal dendrites of CA1 pyramidal neurons that receive excitatory inputs from CA2 pyramidal neurons.^30^ We observed an increased number (135.5%, *p* = 0.0192) and intensity (115.4%, *p* = 0.004) of VGluT1 puncta in CA1 *stratum oriens* of secretome-treated slices when compared to control slices (Figures 2I and 2J). Thus, HUCPVC secretome is capable of inducing presynaptic differentiation in an *ex vivo* model that closely reproduces most anatomical features of the hippocampus *in vivo*.

### Axonal-specific stimulation with HUCPVC secretome induces presynaptic differentiation in CNS neurons

**Figure 3:**
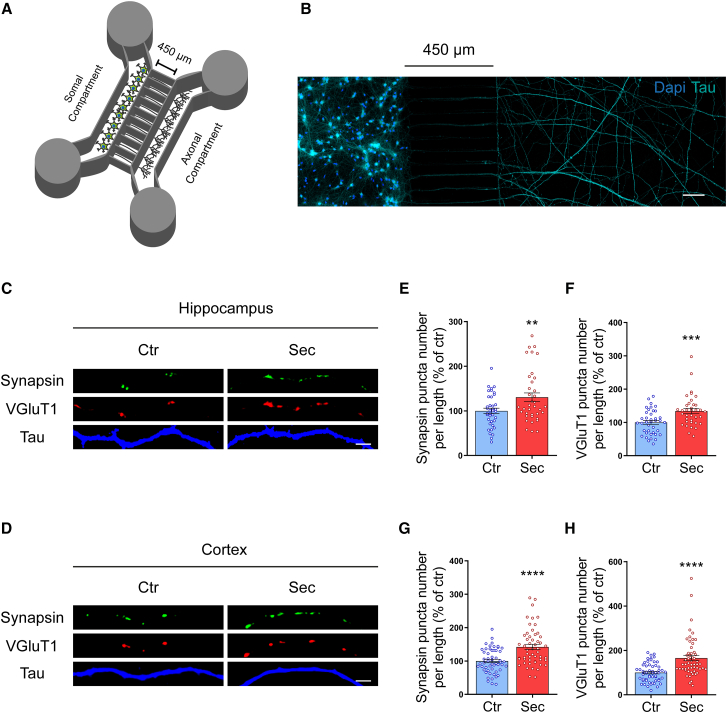
Axonal-specific stimulation with HUCPVC secretome induces formation of new presynaptic sites in CNS neurons (A) Schematic representation of microfluidic chambers. This system consists of a moulded PDMS chamber placed against a glass coverslip, and it is composed by a somal and an axonal compartment. Both compartments are separated by a set of microgrooves (450 μm long, 10 μm wide). The height difference between microgrooves (3 μm) and compartments (100 μm) combined with a minimal volume difference between the two sides leads to a fluidic isolation between the two compartments. (B) Representative image of hippocampal neurons cultured in microfluidic devices and immunostained for tau (cyan) and stained for DNA (blue). Image shows that cell bodies were restricted to the somal compartment while axons crossed the microgrooves and extended throughout the entire surface of the axonal compartment. Thus, microfluidic chambers allow the physical isolation of distal axons and their specific manipulation without somal or dendritic contribution. Contiguous images were taken from an area of the microfluidic chamber using an AxioObserver Z1 fluorescent microscope with a PlanApochromat 20x objective and assembled into a single image using ZEN 2011 software. Scale bars 100 μm. (C and D) Effect of local application of HUCPVC secretome in presynaptic assembly. At DIV7, hippocampal (C) and cortical (D) axons present in the axonal compartment were stimulated for 6 h with secretome. Presynaptic differentiation was assessed by immunocytochemistry using an antibody against the synaptic vesicle markers synapsin (green) or VGluT1 (red); axons were identified using an antibody against tau (blue). Images were acquired from random axons using an AxioObserver Z1 fluorescent microscope with a PlanApochromat 63x oil objective. Scale bars, 2.5 μm. (E–H) Quantification of synapsin (E and G) and VGluT1 (F and H) puncta number per axonal length. Results show that local application of secretome for 6 h significantly increases the number of synapsin and VGluT1 puncta, demonstrating that axonal-specific stimulation with secretome induces the formation of new presynaptic sites in CNS neurons. Puncta analysis was performed with ImageJ 1.45e software. Results are expressed as % of control. Bars represent the mean ± SEM of 38 (Ctr) or 37 (Sec) images from randomly selected areas of three independent cultures (E and F) or the mean ± SEM of 54 images from randomly selected areas of four independent cultures (G and H); (E) ∗∗ represents *p* = 0.0087; (F) ∗∗∗ represents *p* = 0.0007; (G) ∗∗∗∗ represents *p* < 0.0001; and (H) ∗∗∗∗ represents *p* < 0.0001 by unpaired t test when compared to Ctr.

We next asked whether HUCPVC secretome acts locally in distal axons regulating presynaptic differentiation. In order to better mimic the spatial organization and segregation between neuronal compartments in the living organism, we cultured cortical and hippocampal neurons in microfluidic chambers. These devices allow the physical and fluidic isolation of distal axons without any contamination of soma or dendrites.^31^^,^^32^^,^^33^ The microfluidic chambers used in this work are composed by a somal and an axonal compartment separated by a set of small channels, the microgrooves (Figure 3A). Cell bodies are restricted to the somal compartment while axons cross the 450 μm long microgrooves to the axonal compartment and establish a complex network of axonal processes (Figure 3B). Due to their shorter length, dendrites do not reach the axonal compartment. A slight volume difference (30 μL) between the somal and the axonal compartment accompanied by the high fluidic resistance of the microgrooves ensures the fluidic isolation between the two sides.

Hippocampal and cortical neurons were grown in microfluidic chambers until DIV7. After a 24-h starving period, in which the axons were kept in neurobasal medium containing only glutamine and penicillin/streptomycin, HUCPVC secretome was applied to the axonal compartment for 6 h. Presynaptic assembly was evaluated as described before (Figures 3C and 3D). Treatment of hippocampal axons with the secretome significantly increased the number of synapsin (130.5%, *p* = 0.0087) and VGluT1 (134.2%, *p* = 0.0007) puncta per axon length, when compared to control axons (Figures 3E and 3F). Moreover, axonal-specific stimulation with the secretome also promoted an increase in synapsin (142.2%, *p* < 0.0001) and VGluT1 (165.4, *p* < 0.0001) puncta number per axon length in cortical axons (Figures 3G and 3H), when compared to control condition. Thus, these observations reveal that HUCPVC secretome can act locally in distal axons to induce presynaptic differentiation.

### HUCPVC secretome induces axodendritic synapse formation in hippocampal neurons

**Figure 4:**
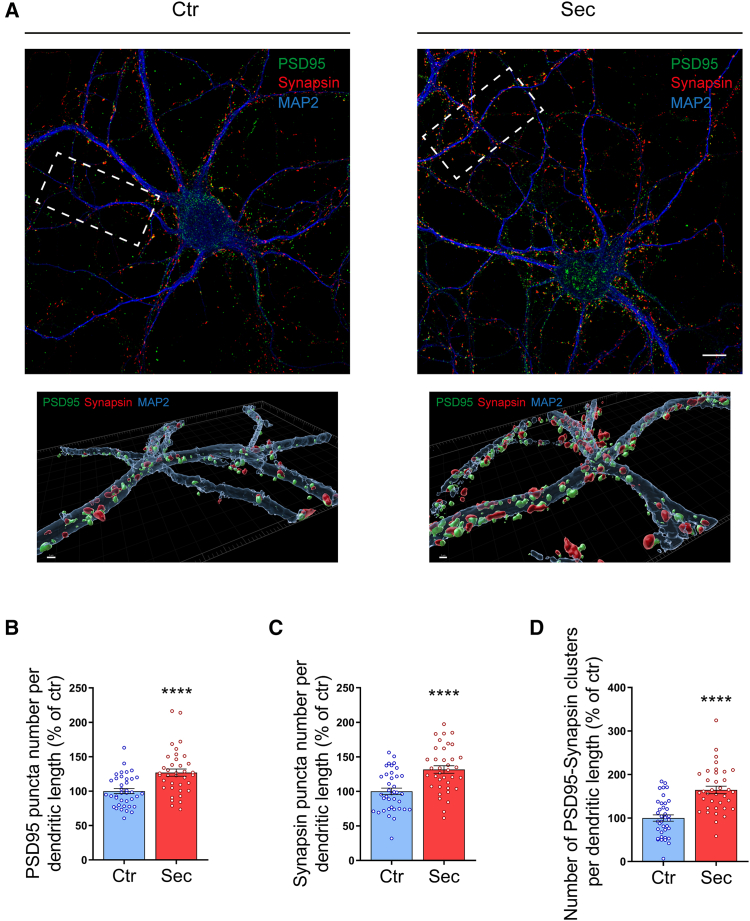
HUCPVC secretome induces axodendritic synapse formation in hippocampal neurons (A) Effect of HUCPVC secretome in the formation of axodendritic synaptic contacts. At DIV14, hippocampal neurons were stimulated for 6 h with secretome. Synapse formation was evaluated by immunocytochemistry using an antibody against the postsynaptic marker PSD95 (green) and the presynaptic marker synapsin (red). Dendrites were visualized using an antibody against MAP2 (blue). Images were acquired from random neurons using a Carl Zeiss LSM 710 confocal microscope with a PlanApochromat 63x oil objective. A 3D reconstruction of the synaptic puncta across the dendrites was done using the Imaris software. Scale bars, 10 μm for the full images and 1 μm for the 3D images. (B–D) Quantification of PSD95 (B) and synapsin (C) puncta number or colocalized PSD95-synapsin clusters (D) per dendritic length. Results show that global application of secretome for 6 h significantly increases the number of colocalized PSD95-synapsin clusters and the number of PSD95 and synapsin puncta alone throughout the dendrites, demonstrating that treatment with the secretome promotes axodendritic synapse formation in hippocampal neurons. Puncta analysis was performed with ImageJ 1.45e software. Results are expressed as % of control. Bars represent the mean ± SEM of 38 (Ctr) or 37 (Sec) images from randomly selected areas of three independent cultures; (B) ∗∗∗∗ represents *p* < 0.0001; (C) ∗∗∗∗ represents *p* < 0.0001; and (D) ∗∗∗∗ represents *p* < 0.0001 by unpaired t test when compared to Ctr.

Through the action of soluble synaptogenic molecules, presynaptic sites are generated in predefined axonal locations.^34^ These orphan presynapses will be the preferential sites for later establishment of axodendritic synapses.^35^ Taking this into consideration, we next asked whether the observed increase in presynaptic sites induced by HUCPVC secretome correlated with an increase in the number of axodendritic synapses. To address this question, we used low-density hippocampal cultures that allow the visualization and isolation of dendritic processes (Figure 4A). At DIV14, hippocampal neurons were treated with HUCPVC secretome for 6 h. The formation of axodendritic synapses was assessed by immunostaining the neurons against the postsynaptic marker PSD95 and the presynaptic marker synapsin. MAP2 immunostaining was used as a dendritic marker. After reconstructing dendritic structures and synaptic puncta in 3D via Imaris (Figure 4A), we observed that secretome stimulation increased the number of synaptic clusters (PSD95 puncta that colocalizes with synapsin puncta) per dendritic length (164.5%, *p* < 0.0001), as well as the individual number of both PSD95 puncta (126.9%, *p* < 0.0001) and synapsin puncta (131.7%, *p* < 0.0001) per dendritic length, when compared to control neurons (Figure 4). These observations demonstrate that HUCPVC secretome enhances axodendritic synapse formation in hippocampal neurons, corroborating the results obtained in isolated axons.

### HUCPVC secretome enhances synaptic activity in hippocampal neurons

**Figure 5:**
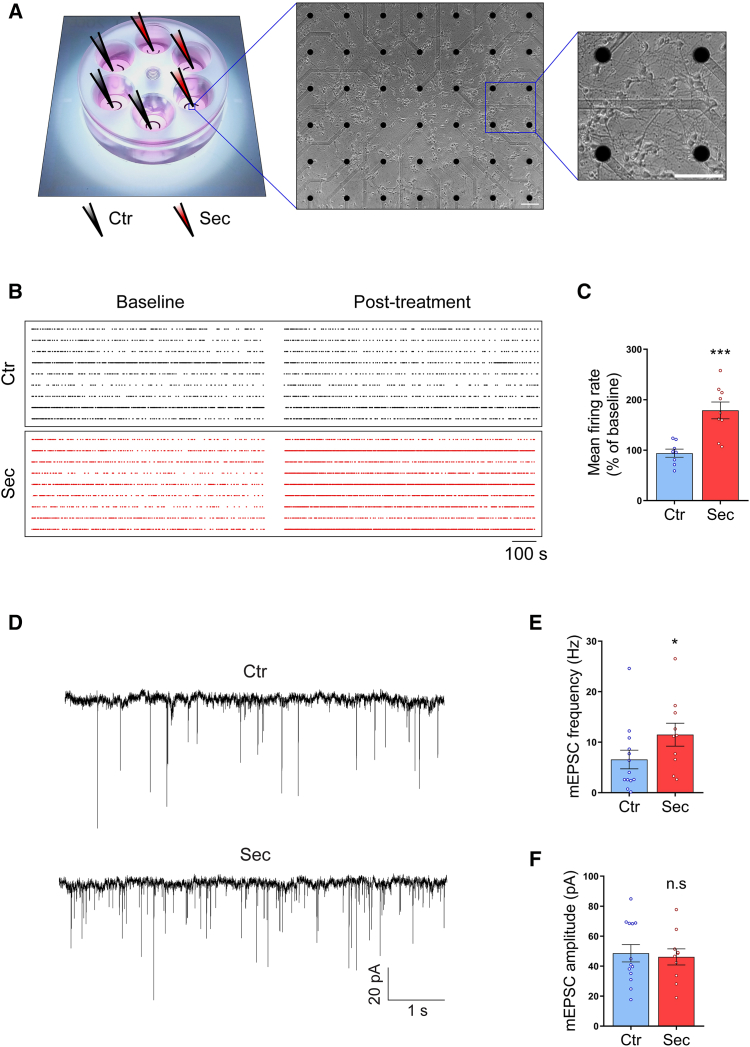
HUCPVC secretome enhances synaptic activity in hippocampal neurons (A) Schematic representation of a 6-well multi-electrode array (MEA) chip. Each well allows the recording of a unique neuronal culture through a total of 42 electrodes distributed in a 6 × 7 grid and a pitch of 200 μm. Image insets show a phase-contrast microscopy image of a typical hippocampal culture at DIV7. Images were obtained with a 10× objective (0.25 NA, Ph1). Scale bars, 100 μm. (B) Raster plots of 30 min of neuronal activity. Each row corresponds to the spike raster plot of an active electrode (firing rate >0.1 Hz) either from control (black dots) or cultures treated with HUCPVC secretome (red dots). At DIV7, hippocampal neurons were challenged with the secretome for 6 h. Treatment-induced changes in neuronal activity were assessed by comparing the mean firing rate (MFR) of the cultures immediately before (baseline) and after stimulation (posttreatment). (C) Mean firing rate of each culture. Results show that secretome stimulation for 6 h significantly increases the firing rate of hippocampal cultures, indicating that HUCPVC secretome enhances the spontaneous activity of hippocampal neurons. The MFR of each culture was calculated in MATLAB 2020a. Bars represent the mean ± SEM of eight wells (Ctr) and nine wells (Sec) from three independent cultures; (C) ∗∗∗ represents *p* = 0.0005 by unpaired t test when compared to Ctr. (D) Representative traces of mEPSCs recorded from a control hippocampal neuron (upper trace) and a neuron treated with HUCPVC secretome (lower trace). At DIV8, neurons were stimulated for 6 h with secretome. The activity of excitatory synapses was evaluated by recording mEPSCs, selectively mediated by AMPA receptors. (E and F) Quantification of mEPSC frequency (E) and amplitude (F). Results show that HUCPVC secretome significantly increases the mEPSC frequency, with no effect on their amplitude, indicating that HUCPVC secretome promotes an increase in excitatory transmission of hippocampal neurons. The recordings were analyzed in ClampFit. Bars represent the mean ± SEM of 13 (Ctr) or 10 (Sec) recordings from three independent cultures; (E) ∗ represents *p* = 0.0358 and (F) n.s represents “not significant” by Mann-Whitney unpaired t test when compared to Ctr.

We next sought to investigate if the new synapses induced by HUCPVC secretome were functional. For this purpose, we measured the spontaneous network activity of hippocampal neurons grown on 6-well MEA chips (Figure 5A). At DIV7, the basal activity of each culture was recorded over a period of 30 min, before a 6-h incubation with HUCPVC secretome. Fresh neurobasal medium was added to control neurons. Afterward, new recordings of 30 min were performed, and the difference in the mean firing rate (MFR) between the baseline and posttreatment recordings was calculated to evaluate possible alterations in the network activity as a result of the treatment (Figure 5B). The neuronal firing rate was significantly higher in the secretome-treated cultures (178.9%, *p* = 0.0005), when compared to control cultures (Figure 5C), indicating that HUCPVC secretome enhances the spontaneous network activity of hippocampal neurons.

To investigate if the observed increase in excitatory synapses after treatment with HUCPVC secretome correlates with an increase in excitatory neurotransmission, we measured miniature excitatory postsynaptic currents (mEPSCs) by whole-cell voltage clamp. At DIV8, hippocampal neurons were stimulated with HUCPVC secretome for 6 h. Afterward, mEPSCs selectively mediated by AMPA receptors were recorded from both control and secretome-treated neurons (Figure 5D). Stimulation of hippocampal neurons with the secretome significantly increased the frequency of mEPSCs (Figure 5E), without changing their amplitude (Figure 5F). The change in frequency, but not amplitude, suggests a synaptogenic effect of HUCPVC secretome that leads to an increased number of active synapses without changing synaptic transmission at the single synapse level. Overall, these results indicate that secretome administration promotes excitatory synaptic transmission by increasing the number of active synapses, thereby functionally validating the results obtained by immunocytochemistry.

### Thrombospondin-1 is the main synaptogenic molecule in HUCPVC secretome

**Figure 6:**
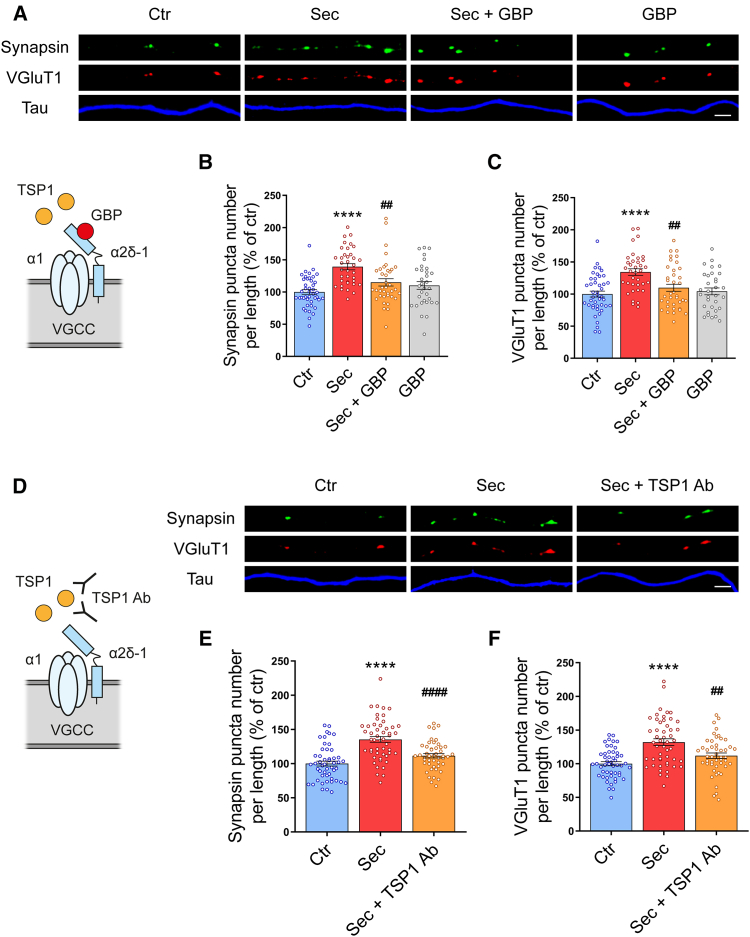
Thrombospondin-1 is the major synaptogenic factor in HUCPVC secretome (A) Gabapentin (GBP), the high-affinity ligand for α2δ-1, inhibits HUCPVC secretome-induced presynaptic differentiation. At DIV8, hippocampal neurons were treated with 32 μM Gbp for 20 min prior to secretome stimulation. The formation of presynaptic clusters was evaluated by immunocytochemistry using an antibody against synapsin (green) or VGluT1 (red). Distal axons were identified using an antibody against tau (blue). Images were acquired from random axons using an AxioObserver Z1 fluorescent microscope with a PlanApochromat 63× oil objective. Scale bars, 2.5 μm. (B and C) Quantitative data of the number of synapsin (B) and VGluT1 (C) clusters per axonal length after treatment with GBP. Results show that GBP prevents the increase in synapsin and VGluT1 puncta number induced by HUCPVC secretome, indicating that the synaptogenic effect of HUCPVC secretome relays on the thrombospondin receptor α2δ-1. Puncta analysis was performed with ImageJ 1.45e software. Results are expressed as % of control. Bars represent the mean ± SEM of 43 (Ctr), 36 (Sec), 37 (Sec+GBP), or 32 (GBP) images from randomly selected areas of three independent cultures; (B) ∗∗∗∗ represents *p* < 0.0001 when compared to Ctr, and ## represents *p* = 0.0029 when compared to Sec; (C) ∗∗∗∗ represents *p* < 0.0001 when compared to Ctr, and ## represents *p* = 0.0043 when compared to Sec. Statistical significance by one-way ANOVA followed by Bonferroni’s multiple comparisons test. (D) Effect of thrombospondin-1 (TSP1) neutralization on HUCPVC secretome synaptogenic activity. At DIV8, hippocampal neurons were stimulated with HUCPVC secretome pre-treated with 10 μg/mL of a neutralizing antibody against TSP1. Images were acquired from random axons using an AxioObserver Z1 fluorescent microscope with a PlanApochromat 63x oil objective. The scale bar is 2.5 μm. (E and F) Quantification of the effect of a monoclonal anti-TSP1 antibody on HUCPVC secretome-induced presynaptic differentiation. Treatment of hippocampal neurons with the secretome for 6 h significantly increases the number of synapsin (E) and VGluT1 (F) puncta per axonal length. This increase, however, is not observed when the secretome is pre-treated with a neutralizing antibody to TSP1, indicating that TSP1 is the main synaptogenic molecule in HUCPVC secretome. Puncta analysis was performed with ImageJ 1.45e software. Results are expressed as % of control. Bars represent the mean ± SEM of 50 (Ctr and Sec) or 47 (Sec + TSP1 Ab) images from randomly selected areas of 4 independent cultures; (E) ∗∗∗∗ represents *p* < 0.0001 when compared to Ctr and #### represents *p* < 0.0001 when compared to Sec; (F) ∗∗∗∗ represents *p* < 0.0001 when compared to Ctr and ## represents *p* = 0.0021 when compared to Sec. Statistical significance by one-way ANOVA followed by Bonferroni’s multiple comparisons test.

HUCPVC secretome presents on its composition a wide range of molecules with different properties that can underlie the observed effects on synapse formation. Proteins with documented roles in the nervous system development such as BDNF, NGF, GDNF, pigment-epithelium-derived factor (PEDF), glia-derived nexin (GDN), IL-6, semaphorin 7A (Sema7A), or TSP1 were identified in HUCPVC secretome.^10^^,^^15^^,^^36^ Among these, TSP1 stands out as a strong candidate since it is a well-known synaptogenesis inducer.^24^^,^^25^^,^^37^ TSPs are large oligomeric extracellular matrix proteins secreted by astrocytes that induce excitatory synaptogenesis in different types of neurons through the calcium channel subunit α2δ-1.^25^^,^^26^^,^^37^^,^^38^ To test whether TSPs mediate HUCPVC secretome synaptogenic activity, we used the pharmacological agent gabapentin (GBP) (Figure 6A). GBP was previously demonstrated to block the synaptogenic effect of TSPs by binding to α2δ-1.^25^^,^^38^ Hippocampal neurons were treated with 32 μM GBP for 20 min prior to secretome stimulation, and presynaptic assembly was assessed by immunocytochemistry as before. As observed previously, HUCPVC secretome significantly increased the number of synapsin (139.3%, *p* < 0.0001) and VGluT1 (134.2%, *p* < 0.0001) puncta per axon length, when compared to control. However, this effect was prevented in neurons pre-treated with GBP (synapsin, 115%, *p* = 0.0029 and VGluT1, 109.8%, *p* = 0.0043 when compared to secretome-treated neurons). GBP alone did not alter the number of presynaptic clusters (Figures 6B and 6C). These results suggest that TSPs are key molecules involved in HUCPVC secretome synaptogenic activity.

To confirm the involvement of TSP1 in HUCPVC secretome-induced presynaptic assembly, we used a neutralizing antibody (Figure 6D). TSP’s synaptogenic activity is linked to their epidermal growth factor (EGF)-like domains, common to all TSP isoforms. They use their EGF-like domains to interact directly with the Von Willebrand factor A (VWF-A) domain of α2δ-1, inducing synaptogenesis.^25^ Monoclonal antibodies against the second or third EGF-like repeats blocked the synaptogenic effect of TSPs.^25^ Taking this into consideration, we pre-treated HUCPVC secretome with a monoclonal antibody against the third EGF-like repeat of TSP1 (Clone A4.1)^39^ for 20 min at 37°C. Presynaptic differentiation was evaluated by immunocytochemistry as described previously. As expected, HUCPVC secretome promoted an increase in synapsin (135.4%, *p* < 0.0001) and VGluT1 (132%, *p* < 0.0001) puncta number per axon length when compared to control. The secretome pre-treated with the anti-TSP1 antibody was unable to reproduce these effects (synapsin, 111.4%, *p* < 0.0001 and VGluT1, 111.9%, *p* = 0.0021 when compared to secretome-treated neurons) (Figures 6E and 6F). Together, these data indicate that HUCPVCsecretome-induced synaptogenesis is mediated by TSP1 through interaction with the α2δ-1 receptor.

### HUCPVC secretome induces excitatory presynaptic differentiation in human cortical brain organoids

**Figure 7:**
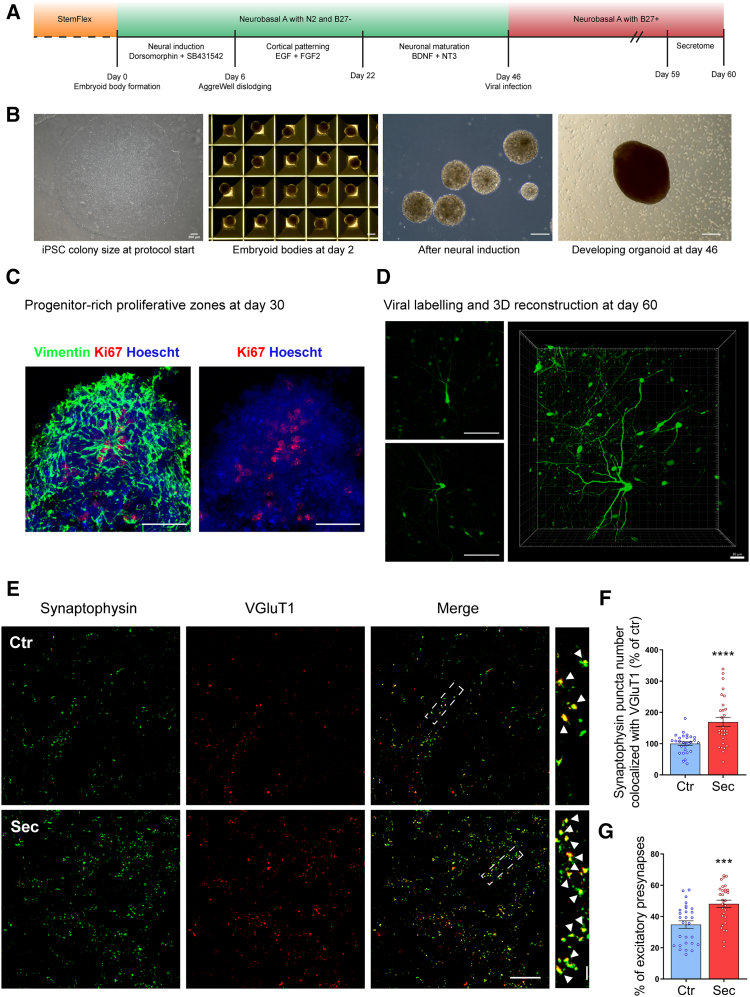
HUCPVC secretome promotes excitatory presynaptic differentiation in human cortical cerebral organoids (A and B) Differentiation timeline of the region-specific cortical organoids used in this study. Embryoid bodies (EB) were generated from iPSCs using micro-patterning wells. The inverted pyramid architecture of each micro-well facilitates spontaneous cell aggregation. Following EB formation, cells underwent neural ectoderm induction through dual SMAD pathway inhibition (Dorsomorphin and SB-431542). At day 46 post-EB formation, organoids are considered self-sufficient, marking the chosen timepoint for viral infection labeling of neurons using AAV9-hSYN-EGFP. At day 59 post-EB formation, secretome was incubated for a 24h period, and the organoids collected. Scale bars: 200 μm. (C) Brain organoids recapitulate key aspects of neurodevelopment, exhibiting radial arrangements of proliferating neural progenitor cells. Scale bars: 100 μm. (D) The progenitors rapidly develop into interconnected neuronal networks as evidenced by prominent AAV9-hSYN-EGFP expression. Scale bars: left-100 μm and right-30 μm. (E) Synaptogenic effect of HUCPVC secretome on human cortical brain organoids. Representative images of organoid sections immunostained for the presynaptic markers synaptophysin (green) and VGluT1 (red). Images were acquired using a Carl Zeiss LSM 710 confocal microscope with a Plan-Apochromat 63x oil objective. The scale bar is 20 μm for the full images and 2.5 μm for the insets. (F and G) Quantitative data on the number of synaptophysin puncta colocalized with VGluT1 per area (F) and the percentage of excitatory presynapses within the organoid sections (G). Treatment of the organoids with the secretome for 24 h significantly increases the number of excitatory presynaptic sites. Puncta analysis was performed with ICY software. Bars represent the mean ± SEM of 28 (Ctr) or 29 (Sec) images (2–3 images per section/3–4 sections per organoid) of 3 organoids per condition; (F) ∗∗∗∗ represents *p* < 0.0001 and (G) ∗∗∗ represents *p* = 0.0002 by unpaired t-test when compared to Ctr.

To evaluate the ability of the HUCPVC secretome to induce synaptogenesis in human neurons and thereby strengthen its translational relevance, we employed human cortical cerebral organoids (Figure 7). In this model, human neurons are generated throughout time in a 3D configuration, mirroring the cellular environment of the developing nervous system. To achieve this, human iPSCs were aggregated into an embryoid body (EB) (Figure 7B), a structure that closely resembles the human blastocyst. Cells were then guided to differentiate toward a neural ectoderm fate through double inhibition of the SMAD pathway using dorsomorphin and SB-431542, with neural rosettes becoming apparent within 6 days following neural induction (Figure 7B). These structures are initially reminiscent of the embryonic neural tube, where neural progenitors organize in a radial manner and differentiate outward, which can be observed with Ki67 and vimentin staining (Figure 7C). The neural progenitors then stratify, generating layers of neurons with diverse identities akin to the human cerebral cortex. To target and visualize mature neurons, organoids were transduced on day 46 with a virus expressing EGFP under the human synapsin promoter (AAV9-hSYN-EGFP). Thirteen days posttransduction, organoids were incubated with the secretome in maintenance media and collected 24 h later. EGFP labeling was used to select areas within the organoid containing mature neurons integrated into neuronal networks for synaptic puncta analysis (Figure 7D). Presynaptic differentiation was accessed by immunostaining the organoids against the SV markers synaptophysin and VGluT1 (Figure 7E). Secretome treatment increased the number of puncta showing synaptophysin/VGluT1 colocalization per area (169,1%, *p* < 0.0001) when compared to the control (Figure 7F). The percentage of excitatory presynapses, given by the number of puncta showing synaptophysin/VGluT1 colocalization divided by the total number of synaptophysin puncta, also increased in the organoids incubated with the secretome (48,1%, *p* = 0.0002) when compared to control organoids (34.8%) (Figure 7G). These findings indicate that HUCPVC secretome is capable of inducing excitatory presynaptic differentiation in human neurons.

## Discussion

In this work, we demonstrate that HUCPVC secretome stimulates the formation of new synapses in CNS neurons. To date, several studies have demonstrated that MSC secretome promotes axonal outgrowth.^10^^,^^12^^,^^16^ However, it remained unknown whether MSC secretome can also influence other phases of axonal development. Our results indicate that HUCPVC secretome promotes axonal branching in cortical and hippocampal neurons (Figure 1). Axonal branching is a crucial event for the establishment of functional synaptic connectivity. To integrate information from divergent regions of the nervous system, each axon must extend multiple branches to communicate with several postsynaptic partners.^27^ Moreover, in several regions of the mammalian brain, axons extend beyond their target region, and it is the branches that arise interstitially from the axon shaft that form connections with the appropriate postsynaptic targets.^40^ Once the axons reach their target region, synapse formation is triggered either by *trans*-synaptic adhesion complexes or by secreted soluble molecules.^18^ These molecules induce the clustering of presynaptic material along the axons, establishing the sites where new synapses will be formed. Notably, we observed an increase in the number of presynaptic clusters in both cortical and hippocampal neurons treated with HUCPVC secretome (Figure 2). A similar synaptogenic effect was also observed in hippocampal organotypic slices stimulated with the secretome (Figure 2), a model that closely resembles the physiological environment. This finding indicates that the synaptogenic activity of HUCPVC secretome is not influenced by the presence of glial cells, hinting that the secretome may be able to replicate these effects *in vivo*. In order to mimic the physiological environment *in vitro*, in which distal axons are at a considerable distance from the cell bodies and dendrites, and to locally apply the secretome to the axons, we used microfluidic chambers (Figure 3). Local application of HUCPVC secretome to distal axons present in the axonal compartment of these devices is able to induce SV clustering (Figure 3), indicating that the secretome is able to act locally at the axonal level to activate the machinery involved in presynaptic differentiation. Previously, we also showed that a localized application of HUCPVC secretome to distal axons induces robust axonal outgrowth.^10^ In conjunction with the present study, we demonstrate that axonal-specific administration of secretome is sufficient to promote both axonal outgrowth and presynaptic differentiation. This is an important finding in the axonal regeneration field since a new therapeutic approach based on the use of MSC secretome must rely on a local application to the injured axons and not to the entire neuron.

It is widely accepted that the developing axon has an intrinsic capacity to generate orphan presynaptic sites in specific locations before any contact with its postsynaptic partner. Secreted factors such as fibroblast growth factors, Wnts, neurotrophic factors, and TSPs have been described to play a fundamental role in this process.^18^^,^^34^^,^^41^ The orphan presynaptic sites are capable of performing SV fusion and recycling events^42^ but also constitute the location where a future axodendritic synapse will be formed.^35^ In line with the observed increase in presynaptic sites, treatment with HUCPVC secretome also increases the number of presynaptic clusters formed onto dendrites and the number of axodendritic synapses (Figure 4).

Our study also reveals that HUCPVC secretome enhances synaptic activity in cultured hippocampal neurons (Figure 5). This demonstrates that HUCPVC secretome promotes the formation of functional synapses, which are able to fuse neurotransmitter-filled synaptic vesicles upon depolarization and, consequently, promote an increase in synaptic activity. This is in line with the findings of Kim and colleagues, who observed an increase in synaptic transmission in hippocampal neurons after being co-culture with human umbilical-cord-blood-derived MSCs.^43^ Contrary to our results, Mauri and colleagues did not observe significant differences in excitatory synapses and neurotransmission in hippocampal neurons co-cultured with rat bone-marrow-derived MSCs.^22^ One possible explanation for these results is that MSCs from different sources may have different secretion profiles. In fact, by performing a comparative proteomics-based analysis through mass spectrometry on the secretome of human MSCs derived from the bone marrow, adipose tissue, and umbilical cord, Pires and colleagues identified a specific proteomic profile for each population.^15^ Several proteins with neurotrophic, neurogenic, axon guidance, and neuroprotective actions were differently expressed by distinct populations of MSC. Therefore, depending on the tissue of origin, the therapeutic outcome of MSC secretome may be different.

The TSP family of extracellular matrix proteins was the first synaptogenic molecules secreted by astrocytes to be identified. TSP1 and TSP2 are produced by astrocytes during early postnatal development and were initially described to induce synapse formation in retinal ganglion cells (RGCs) by Christopherson and colleagues.^24^ Subsequently, similar effects were described in dorsal root ganglia (DRG) neurons and hippocampal neurons.^26^^,^^37^^,^^38^ Transgenic animals lacking TSP1 and TSP2 also have a significant decrease in the number of excitatory synapses in the cortex.^24^ Their synaptogenic effect is mediated by the voltage-gated calcium channel subunit α2δ-1, which is expressed by neurons both pre- and postsynaptically throughout the central and peripheral nervous systems.^44^ α2δ-1 null mice show decreased synaptic density in the cortex^26^ and in the CA1 area of the hippocampus,^45^ reinforcing the role of this calcium channel subunit in TSPs-induced synapse formation. Moreover, the anti-epileptic and analgesic drug GBP, a high affinity ligand for α2δ-1, completely inhibits TSPs synaptogenic activity both *in vitro* and *in vivo*.^25^^,^^38^ Remarkably, we observed that GBP treatment prevents HUCPVC secretome-induced presynaptic assembly in hippocampal neurons (Figure 6), indicating that TSPs mediate HUCPVC secretome synaptogenic effect. All TSP isoforms (TSP1-TSP5) can induce synapse formation.^25^ However, TSP1 was the only isoform identified in HUCPVC secretome by mass spectrometry,^15^ and inhibition of TSP1 activity using neutralizing antibodies reproduces the effects of GBP treatment (Figure 6), demonstrating that TSP1 is the main synaptogenic molecule secreted by HUCPVC.

Our results indicate that TSP1 acts as a presynaptic differentiation molecule, acting through presynaptic α2δ-1 to induce SVs clustering (Figure 6). This is in line with the findings of Yu and colleagues, who showed that TSP4 interacts with presynaptic, but not postsynaptic, α2δ-1 to induce excitatory synaptogenesis in DRG neurons.^38^ This presynaptic effect of TSP/α2δ-1 can be modulated by the activation of presynaptic NMDA receptors (NMDARs). Through its C-terminal domain, α2δ-1 forms a heteromeric complex with NMDARs, promoting their synaptic targeting and activity.^46^ Activation of NMDARs seems to be a required step for presynaptic differentiation. Treatment of cortical neurons with the selective NMDAR antagonist D-2-amino-5-phosphonovaleric acid (APV) compromises the accumulation of several SV and active zone proteins at developing presynaptic terminals. Moreover, in presynaptic terminals being formed on non-neuronal cells that lack NMDARs, clustering of presynaptic material was also reduced upon inhibition of NMDAR activity, demonstrating the presynaptic necessity of NMDARs for SV clustering.^47^ In addition, the astrocyte-derived TGF-β1 also promotes synapse formation in a manner dependent on NMDAR activity.^48^ Based on these findings, we can speculate that TSP binding to the presynaptic α2δ-1 recruits and activates NMDAR on the axonal surface, which then triggers downstream signaling cascades that enhance the clustering of presynaptic material. Contrary to these findings, Risher and colleagues revealed that postsynaptic α2δ-1 is sufficient for TSP2-induced synaptogenesis in the cortex. Binding of TSP2 to α2δ-1 in dendritic filopodia activates the small Rho GTPase, Rac1, that promotes synaptic development and spinogenesis.^26^ However, the mechanism by which postsynaptic α2δ-1 induces the differentiation of the presynaptic site upon TSP2 binding remains to be elucidated. Further studies should be performed to characterize the precise synaptogenic role of pre- and postsynaptic α2δ-1 and the molecular mechanisms involved.

Several approaches have succeeded in promoting axon regeneration after CNS injury. However, behavioral improvements are often modest or even neutral.^49^^,^^50^ To achieve functional recovery, the injured axon must be able to re-establish the synaptic contacts that were lost. Therefore, future strategies to improve the regenerative capacity of injured CNS axons must focus not only on axon outgrowth but also on synapse formation. Although the mechanisms involved in postinjury synapse formation are not completely clarified, several reports suggest that the same synaptogenic factors that operate during development may be involved in this process.^19^^,^^20^^,^^21^ For instance, TSP1, the main synaptogenic factor in HUCPVC secretome, has been described to induce postinjury synapse formation in different injury paradigms.^21^^,^^51^ Furthermore, HUCPVC secretome is also capable of inducing synaptogenesis in human cortical brain organoids (Figure 7), underscoring its translational potential for the treatment of traumatic CNS disorders. These findings suggest that HUCPVC secretome may be capable of reproducing its synaptogenic effects in injured neurons. Further studies should be performed to confirm this hypothesis.

Altogether, we revealed that HUCPVC secretome can influence all the main phases of axonal development leading up to increased synapse formation, making it a promising therapeutic strategy to tackle SCI and other CNS diseases.

### Limitations of the study

Although this study provides compelling evidence of the involvement of TSP1 in HUCPVC secretome synaptogenic activity, future studies employing animal models of axonal injury will be important to assess the secretome potential as a cell-free therapy for traumatic CNS disorders and its ability to promote synapse formation following injury.

## Resource availability

### Lead contact

Further information and requests for resources and reagents should be directed to and will be fulfilled by the lead contact, Ramiro D. Almeida (ramirodalmeida@gmail.com).

### Materials availability

This study did not generate new unique reagents.

### Data and code availability

• Microscopy and electrophysiology data reported in this paper will be shared by the lead contact upon request.
• This paper does not report original code.
• Any additional information required to reanalyze the data reported in this paper is available from the lead contact upon request.

## Acknowledgments

This work was supported by “la Caixa” Foundation (ID 100010434) and FCT-Fundação para a Ciência e a Tecnologia, I.P., under the agreement LCF/PR/HP20/52300001, Angelman Syndrome Alliance Grant 2024 and by 10.13039/501100001871FCT, I.P., under projects UID 4501- 10.13039/100020794Instituto de Biomedicina, Aveiro, UIDB/04539/2020, UIDP/04539/2020, and LA/P/0058/2020 and through the individual grant SFRH/BD/139368/2018 to D.T.

## Author contributions

D.T. performed the experiments, analyzed data, participated in the experimental strategy design, and wrote the manuscript; L.F.M. and R.O.C. assisted with the cell cultures, protocol optimization, and data analysis; H.S. and J.P. generated the human cortical brain organoids and assisted with protocol optimization; S.C.S. carried out the secretome collection and concentration; J.L.A. performed the HUCPVC phenotypic and functional characterization; M.A. and P.A. performed the multi-electrode arrays experiments; P.S.P. assisted with patch-clamp electrophysiology experiments and contributed to manuscript drafting; C.B.D. and A.J.S. contributed to the study design and R.D.A. conceived and coordinated the study, designed the experimental strategy, and contributed to manuscript drafting. All authors reviewed and approved the manuscript for publication.

## Declaration of interests

The authors declare no competing interests.

## STAR★Methods

### Key resources table

**Table:** 

REAGENT or RESOURCE	SOURCE	IDENTIFIER
**Antibodies**		

Chicken polyclonal anti-Tau	Abcam	Cat# ab75714; RRID: AB_1310734
Chicken polyclonal anti-MAP2	Abcam	Cat# ab5392; RRID: AB_2138153
Mouse monoclonal anti-β-Tubulin III	Sigma-Aldrich	Cat# T8660; RRID: AB_477590
Mouse monoclonal anti-PSD95 (clone 6G6-1C9)	Thermo Fisher Scientific	Cat# MA1-045; RRID: AB_325399
Rabbit polyclonal anti-Synapsin I	Millipore	Cat# AB1543; RRID: AB_2200400
Mouse monoclonal anti-Thrombospondin 1 (clone A4.1)	Santa Cruz Biotechnology	Cat# sc-59886; RRID: AB_793044
Guinea pig polyclonal anti-VGluT1	Millipore	Cat# AB5905; RRID: AB_2301751
Mouse monoclonal anti-VGluT1	Millipore	Cat# MAB5502; RRID: AB_11214451
Rabbit monoclonal anti-Synaptophysin (clone YE269)	Abcam	Cat# ab32127; RRID: AB_2286949
Chicken polyclonal anti-GFP	Abcam	Cat# ab13970; RRID: AB_300798
Rabbit monoclonal anti-Vimentin	Abcam	Cat# ab92547; RRID: AB_10562134
Mouse monoclonal anti-Ki67	BD Biosciences	Cat# 556003; RRID: AB_396287
Mouse monoclonal PE anti-human CD90 (Thy 1) (clone 5E10)	BioLegend	Cat# 328109; RRID: AB_893442
Mouse monoclonal PE/Cyanine7 anti-human CD73 (clone AD2)	BioLegend	Cat# 344009; RRID: AB_2561541
Mouse monoclonal FITC anti-human CD105 (clone 43A3)	BioLegend	Cat# 323204; RRID: AB_755956
Mouse monoclonal Brilliant Violet 605™ anti-human CD45 (clone HI30)	BioLegend	Cat# 304042; RRID: AB_2562106
Mouse monoclonal APC anti-human CD44 (clone BJ18)	BioLegend	Cat# 338805; RRID: AB_1501202
Mouse monoclonal Brilliant Violet 510™ anti-human HLA-DR (clone L243)	BioLegend	Cat# 307645; RRID: AB_2561396
Alexa fluor secondary Ab goat anti-rabbit 488	Thermo Fisher Scientific	Cat# A-11034; RRID: AB_2576217
Alexa fluor secondary Ab goat anti-rabbit 568	Thermo Fisher Scientific	Cat# A-11036; RRID: AB_10563566
Alexa fluor secondary Ab goat anti-chicken 488	Thermo Fisher Scientific	Cat# A-11039; RRID: AB_2534096
Alexa fluor secondary Ab goat anti-chicken 568	Thermo Fisher Scientific	Cat# A-11041; RRID: AB_2534098
Alexa fluor secondary Ab goat anti-chicken 647	Thermo Fisher Scientific	Cat# A-21449; RRID: AB_2535866
Alexa fluor secondary Ab goat anti-guinea pig 488	Jackson Immuno Research Labs	Cat# 106-545-003; RRID: AB_2337438
Alexa fluor secondary Ab goat anti-guinea pig 647	Thermo Fisher Scientific	Cat# A-21450; RRID: AB_2535867
Alexa fluor secondary Ab goat anti-mouse 568	Thermo Fisher Scientific	Cat# A-11031; RRID: AB_144696
Alexa fluor secondary Ab goat anti-mouse 647	Thermo Fisher Scientific	Cat# A-21235; RRID: AB_2535804

**Bacterial and virus strains**		

pAAV-hSyn-EGFP (AAV9)	Addgene	Cat# 50465-AAV9

**Biological samples**		

Human umbilical cord perivascular cells (HUCPVC)	Tissue Regeneration Therapeutics Inc.	N/A
Human iPSC line	Lutz et al.^52^	N/A

**Chemicals, peptides, and recombinant proteins**		

Poly-D-lysine	Sigma-Aldrich	Cat# P7886
Mouse laminin I	Cultrex, Trevigen	Cat# 3400-010-02
Deoxyribonuclease I	Sigma-Aldrich	Cat# DN25
5-fluoro-2′-deoxyuridine thymidylate synthase inhibitor	Sigma-Aldrich	Cat# F0503
StemFlex^TM^ medium	Thermo Fisher Scientific	Cat# A3349401
Essential 6™ medium	Thermo Fisher Scientific	Cat# A1516401
Neurobasal™ Medium	Thermo Fisher Scientific	Cat# 21103049
Neurobasal™-A Medium	Thermo Fisher Scientific	Cat# 10888022
*N*-2 Supplement	Thermo Fisher Scientific	Cat# 17502048
B-27™ supplement	Thermo Fisher Scientific	Cat# 17504044
B-27™ supplement, minus vitamin A	Thermo Fisher Scientific	Cat# 12587010
Gabapentin	Sigma-Aldrich	Cat# 5.08506
ProLong™ gold antifade mountant	Thermo Fisher Scientific	Cat# P36935
Tetrodotoxin	Tocris	Cat# 1078
Bicuculline	Tocris	Cat# 0130
D-2-amino-5-phosphonovaleric acid	Tocris	Cat# 0106
Y-27632	Selleckchem	Cat# S1049
Dorsomorphin	Sigma-Aldrich	Cat# P5499
SB 431542	Tocris	Cat# 1614
XAV 939	Tocris	Cat# 3748
Human EGF, Animal-Free Recombinant Protein	Thermo Fisher Scientific	Cat# AF-100-15
Human FGF-basic (FGF-2/bFGF) (154 aa) Recombinant Protein	Thermo Fisher Scientific	Cat# 100-18B
Human/Mouse/Rat BDNF Recombinant Protein	Thermo Fisher Scientific	Cat# 450-02
Human NT-3 Recombinant Protein	Thermo Fisher Scientific	Cat# 450-03
N^6^,2′-*O*-Dibutyryladenosine 3′,5′-cyclic monophosphate sodium salt	Sigma-Aldrich	Cat# D0627
*cis*-4,7,10,13,16,19-Docosahexaenoic acid	Sigma-Aldrich	Cat# D2534
Alizarin Red S	Sigma-Aldrich	Cat# A5533
Oil Red O	Sigma-Aldrich	Cat# O0625

**Critical commercial assays**		

MesenCult™ adipogenic differentiation kit (human)	STEMCELL technologies	Cat# 05412
MesenCult™ osteogenic differentiation kit (human)	STEMCELL technologies	Cat# 05465

**Experimental models: Organisms/strains**		

Wistar-Han rats	Charles River, Barcelona, Spain	Strain Code 273

**Software and algorithms**		

ImageJ/Fiji	NIH	https://imagej.net/software/fiji/downloads
ICY software	N/A	https://icy.bioimageanalysis.org/
GraphPad Prism 8	GraphPad	https://www.graphpad.com/
Illustrator CC	Adobe	https://www.adobe.com
MATLAB	MathWorks	https://www.mathworks.com/?s_tid=gn_logo
ClampFit	Molecular Devices	https://www.moleculardevices.com/
IMARIS software	Oxford Instruments	https://imaris.oxinst.com/
Endnote	Clarivate analytics	https://www.endnote.com

### Experimental model and study participant details

#### Animals

Wistar-Han female rats (Charles River, Barcelona, Spain) were housed (two per cage) and maintained in a controlled environment at 22°C–24°C with 55% humidity, on a 12 h light/dark cycle and fed with regular rodent’s chow and tap water *ad libitum*. Embryos at embryonic day (E) 17–18 (males and females) were used for primary neuronal cultures. For organotypic slice cultures, postnatal day (P) 5–6 rats (males and females) were used. All manipulations were done after approval from the Center for Neuroscience and Cell Biology (CNC) Animal Welfare Committee and from the Portuguese national authority for animal experimentation, Direção Geral de Veterinária (ORBEA_237_ 2019/28082019), and in accordance with the approved guidelines and regulations on animal care and experimentation stated in the European Union Directive 2010/63/EU. For this study, a total of 36 animals were used.

### Method details

#### Preparation of microfluidic devices

Microfluidic devices consist of a moulded poly-dimethylsiloxane (PDMS) chamber (20 mm × 25 mm) assembled on a glass coverslip.^31^^,^^53^ This system is composed by two compartments, each 1.5 mm wide and 7 mm long, separated by a set of microgrooves (450 μm long, 10 μm wide). The height difference between microgrooves (3 μm) and compartments (100 μm), combined with a minimal volume difference between the two sides (30 μL) leads to a fluidic isolation between the two compartments.

Microfluidic chambers were prepared as described before.^33^^,^^54^ Briefly, PDMS was prepared from a silicon elastomer kit (Sylgard 184; Dow Corning), cured in molds for 4 h at 60 °C, sterilized with 70% ethanol and assembled onto a glass coverslip. Coverslips were previously treated with 65% nitric acid, extensively washed with milliQ H_2_O, rinsed in 70% ethanol, dried and sterilized under UV radiation. Before plating cells, coverslips were coated with 0.1 mg/mL of poly-D-lysine (PDL) (Sigma) overnight at 37 °C and with 2 μg/mL of laminin (Cultrex) for 2 h at 37°C.

#### Preparation of multi-electrode arrays

6-well multi-electrode arrays (MEAs) were air plasma cleaned for 1 min at 15 W for surface hydrophilization. Then, they were sterilized with 70% ethanol, air-dried inside a laminar flow chamber and exposed to UV light for at least 15 min. The MEAs were sequentially coated with 20 μg/mL of PDL (Sigma) and with 5 μg/mL of laminin (Cultrex) at 37°C for at least 1h.

#### Primary neuronal cultures

Primary cultures of rat embryonic cortical and hippocampal neurons were prepared as previously described,^32^ with minor modifications. After dissection, cortices and hippocampi from E17-E18 Wistar rat embryos were incubated with trypsin (0.045%; Gibco) and deoxyribonuclease (0.01% v/v; Sigma) in Hank’s balanced salt solution (HBSS) (5.36 mM KCl, 0.44 mM KH_2_PO_4_, 137 mM NaCl, 4.16 mM NaHCO_3_, 0.34 mM Na_2_HPO_4_.2H_2_O, 5 mM Glucose, 1 mM sodium pyruvate, 10 mM HEPES, 0.001% Phenol red, pH 7.2) for 15 min at 37°C. Afterward, Hank’s solution with trypsin was removed and the cortices and hippocampi were washed with plating medium [Minimum Essential Medium Eagle (MEM, Sigma) with 26.2 mM NaHCO_3_, 25 mM Glucose and 1 mM sodium pyruvate] containing 10% fetal bovine serum (Biochrom) to stop trypsin activity. Then, both tissues were mechanically dissociated in fresh plating medium and cell density determined. Cells were plated on PDL-coated coverslips at a density of 80 x 10^3^ cells/cm^2^ for electrophysiology experiments or 2.5 x 10^4^ cells were plated at the middle of a coverslip for the analysis of axonal branching and presynaptic differentiation. In microfluidic chambers, 7 x 10^4^ cells were plated in the somal compartment. In MEAs, 5 x 10^4^ cells were seeded on each well. Neurons were allowed to attach for 3 h and then the plating medium was replaced by culture medium [Neurobasal medium (Gibco) supplemented with 2% B27 (Gibco), 0.5 mM glutamine (Gibco) and 12.5 Units/ml penicillin together with 12.5 μg/mL streptomycin (Gibco)]. In hippocampal cultures, 25 μM glutamate (Gibco) was added to the culture medium. Cells were maintained at 37°C in a humidified incubator under an atmosphere of 95% air and 5% CO_2_. At DIV 3–4, the mitotic inhibitor 5-fluoro-2′-deoxyuridine thymidylate synthase inhibitor (5-FDU) (10 μM; Sigma) was added to the cultures to reduce contamination with glial cells.

For the analysis of axodendritic synapses, low-density hippocampal cultures were prepared as previously described.^55^ Briefly, hippocampi were dissected from E17-E18 rat embryos and the cells were dissociated as described above, before plating in neuronal plating medium at a final density of 2.4 x 10^3^ cells/cm^2^ on PDL-coated coverslips. After 3 h, coverslips were flipped over an astroglial feeding layer in Neurobasal medium (Gibco) supplemented with 2% B27 (Gibco), 0.5 mM glutamine (Gibco) and 12.5 Units/ml penicillin together with 12.5 μg/mL streptomycin (Gibco) and 25 μM glutamate (Gibco). Neurons grew face down over the astroglial layer but were kept separate from the glial cells by wax dots on the neuronal side of the coverslips. Cells were maintained at 37°C in a humidified incubator under an atmosphere of 95% air and 5% CO_2_. At DIV 3–4, the mitotic inhibitor 5-FDU (10 μM; Sigma) was added to the cultures to prevent overgrowth of glial cells.

#### Organotypic hippocampal slice cultures

Organotypic slice cultures (300 μm thickness) were prepared from postnatal day 5–6 Wistar rats. Briefly, animals were decapitated, and their brains were removed and placed in ice-cold dissection solution (10 mM glucose, 24 mM NaHCO_3_, 234 mM sucrose, 4 mM KCl, 0.5 mM MgCl_2_, 0.7 mM CaCl_2_ and 0.5% phenol red) previously bubbled with carbogen (95% O_2_ and 5% CO_2_). Whole, intact hippocampi were rapidly dissected out and sliced sagitally using a McILWAIN tissue chopper. The slices were then separated in the dissection solution and transferred onto cell culture inserts (Millicell, Millipore), which had been supplied with 1 mL of culture medium [MEM (Sigma) with 20% heat-inactivated horse serum (Gibco), 1 mM L-glutamine (Gibco), 30 mM HEPES, 13 mM glucose, 5.2 mM NaHCO_3_, 2 mM MgSO_4_, 1 mM CaCl_2_, 1 mg/L insulin and 0.0012% ascorbic acid, pH 7.25 and osmolarity of 310–320 mOsM] in a 6-well plate. The cultures were maintained at 37 °C in a humidified incubator under an atmosphere of 95% air and 5% CO_2_. The medium was changed every two days.

#### iPSC culture

The iPSC line used in this study was kindly gifted by T. Boeckers (University of Ulm). The cell line has a female genotype and originated in^52^ as CTRL1. iPSCs were maintained in StemFlex media (Gibco) and seeded onto Corning Matrigel hESC-qualified matrix (Corning), LDEV-free coated 6-multiwell plates. Media was changed every other day. Cells were allowed to reach 80% confluency and split subsequently with an in-house 0.5M EDTA(Na_4_) solution in phosphate buffered saline (PBS).

#### Generation of cortical brain organoids

The generation of region-specific cortical brain organoids was based on the protocol by Miura and colleagues^56^ with some adjustments. Cultured iPSCs with about 80% confluence were detached and dissociated into single cells by incubation with Accutase (STEMCELL Technologies) for about 10 min at 37°C. Following dissociation, 1.5 x 10^6^ cells were resuspended in StemFlex supplemented with 10 μM Y-27632 (Selleckchem) and plated on a 24-well micro-patterning AggreWell800 plate (STEMCELL Technologies). The micro-patterning plate allows the spontaneous aggregation of seeded iPSCs into 300 embryoid bodies (EBs) through 800 μm-wide micro-wells. After a 24h incubation at 37°C/5% CO_2_, EB formation was confirmed, and media was changed to a differentiation-allowing culture media – Essential 6 (Gibco), supplemented with SMAD pathway inhibitors Dorsomorphin (SigmaAldrich) and SB-431542 (Tocris) at 5 μM and 10 μM, respectively. The epigenetic modulator XAV-939 (Tocris) at 1.25 μM was also added to Essential 6 supplemented-media to promote neural induction efficiency. Half media changes were performed daily until day 6 post-EB formation, when EBs were dislodged with a cut pipette tip and recovered via a 37 μm reversible strainer. EBs were transferred to a 6-well flat bottom Ultra-low attachment plate (Corning) containing cortical patterning media consisting of Neurobasal A (Gibco) supplemented with 1% N2 supplement (Gibco), 2% B27 Minus Vitamin A supplement (Gibco), 1% Penicillin-Streptomycin (Gibco), 1% Glutamax (Gibco) and human recombinant Endothelial growth factor (EGF) and Fibroblast growth factor 2 (FGF2) (PeproTech) at 20 ng/mL in order to promote self-renewal of neural progenitors and regionally pattern these cells into cerebral cortex progenitors. A third of the media was changed every other day until day 22. At this timepoint, EGF and FGF2 were replaced by 20 ng/mL recombinant human Brain derived neurotrophic factor (BDNF) (PeproTech), 20 ng/mL recombinant human Neurotrophin 3 (NT3) (PeproTech), 200 μM Ascorbic acid (SigmaAldrich), 50 μM Adenosine 3′,5′-cyclic monophosphate,N^6^,O2′-Dibutyryl-, sodium salt (cAMP) (SigmaAldrich), and 10 μM *cis*-4,7,10,13,16,19-Docosahexaenoic acid (DHA) (SigmaAldrich) in the previously described supplemented Neurobasal A culture media. The human recombinant proteins along with the small molecules promote a neurogenic shift in the progenitor-rich organoid. Time between media changes was then spaced to every two days until day 46.

#### HUCPVC secretome collection and concentration

HUCPVC were harvested from umbilical cords of consenting donors who underwent full-term caesarean sections, following ethical approval from the University of Toronto and University Health Network, Toronto. Cells were isolated and expanded by Tissue Regeneration Therapeutics Inc. (Toronto, Ontario, Canada) using a proprietary protocol and provided at passage 1. HUCPVC from three different donors were used and expanded as described previously.^15^ Briefly, HUCPVC were resuspended in α-MEM medium (Gibco) supplemented with 10% of FBS (Biochrom) and 1% penicillin/streptomycin (Gibco) and plated at a density of 4000 cells/cm^2^. Cells were maintained at 37 °C in a humidified incubator under an atmosphere of 95% air and 5% CO_2_. The medium was changed every 3 days until cells reached confluency. Secretome was collected in the form of conditioned medium from passage 6 HUCPVC. For this purpose, cells were enzymatically dissociated with 0.05% trypsin-EDTA (Invitrogen) for 5 min at 37°C and plated at a density of 4000 cells/cm^2^ in T175 tissue culture flasks. After 3 days, the culture medium was removed, and the cells were washed four times with phosphate-buffered saline (PBS) without Ca^2+^/Mg^2+^ (Millipore) and then two times with Neurobasal A medium (Gibco). Next, neurobasal A medium supplemented with 1% of kanamycin (Invitrogen) was added to the cells and 24 h later, the conditioned medium was collected and 100 times concentrated by centrifugation at 3000*g* at 4°C for 45 min using a 5 kDa cut-off concentrator (Vivaspin20 Sartorius). At the time of secretome collection, cells reach a confluence of approximately 80–85%. The secretome of at least two different donors was used in each one of the experiments described in this paper. Neurobasal A medium containing 1% kanamycin was 100 times concentrated using the same protocol.

#### HUCPVC phenotypic characterization

For phenotypic characterization, single-cell suspensions of HUCPVC were obtained through enzymatic dissociation with 0.05% trypsin–EDTA (Invitrogen) at passage 3. After cell counting, 90 000 cells were transferred to a round-bottom 96-well plate and resuspended in FACS buffer (0.5% bovine serum albumin (BSA) in PBS buffer). Antibodies were added to each well and incubated for 20 min at 4°C. The following antibodies were used: PE anti-human CD90 (Thy1), PE/Cyanine 7 anti-human CD73, FITC anti-human CD105, Brilliant Violet 605 anti-human CD45, APC anti-human CD44 and Brilliant Violet 510 anti-human HLA-DR (all from BioLegend). Cells were then washed twice and resuspended in FACS buffer. Samples were acquired on a 12-colour BD LSRII flow cytometer using the FACS DIVA software (Becton Dickinson). FlowJo software version 10.0.8 (Tree Star) was used to analyze flow cytometer data.

#### HUCPVC’s differentiation potential

Adipogenic and osteogenic differentiation of HUCPVC was performed using MesenCult adipogenic differentiation kit (human) and MesenCult osteogenic differentiation kit (human) (both from STEMCELL Technologies), according to the manufacturer’s instructions. A group of undifferentiated cells was maintained under normal culture conditions as a differentiation control. After 24 days of differentiation, cells were fixed with 4% paraformaldehyde in PBS for 15 min. For osteogenic differentiation, Alizarin Red S (Sigma) was used to stain calcium deposits in osteocytes. For that purpose, cells were incubated with Alizarin Red S for 40 min at room temperature, followed by 3 washes with PBS. Adipogenic differentiation was evaluated by incubating the cells with Oil Red O staining (Sigma) for 30 min at room temperature, followed by 3 washes with PBS. Cells were then imaged at 10x magnification using an Olympus Widefield Inverted Microscope IX53.

#### Secretome experiments

Culture medium of both cortical and hippocampal neurons was replaced by fresh Neurobasal medium containing glutamine and penicillin/streptomycin with or without HUCPVC-conditioned medium. In the case of microfluidic chambers, only the culture medium present in the axonal compartment was replaced by fresh medium, 24 h before addition of HUCPVC-conditioned medium. In low density cultures, only one-third of the medium was replaced by fresh medium with or without HUCPVC-conditioned medium. In all experiments, HUCPVC-conditioned medium was diluted in fresh medium in order to be 2 times concentrated in the neuronal cultures. For synaptic puncta and electrophysiology experiments, neurons were stimulated for 6h with HUCPVC secretome, for axonal branching experiments, neurons were stimulated for 14h. To prevent TSP1 binding to the calcium channel subunit α2δ-1, neurons were treated with 32 μM gabapentin (Sigma) for 20 min at 37°C prior to secretome stimulation. To block TSP1 activity directly, HUCPVC secretome was incubated with 10 μg/mL of a mouse monoclonal IgM antibody against the third EGF-like repeat of TSP1 (Clone A4.1, Santa Cruz Biotechnology) for 20 min at 37°C. Secretome treatment was performed at DIV 7–8, which corresponds to the onset of synaptogenesis in primary hippocampal cultures,^57^ unless otherwise indicated. In organotypic slice cultures, the culture medium was replaced by fresh medium containing HUCPVC secretome or neurobasal A medium (Control) 2 times concentrated. Slices were treated with the secretome at DIV4 for 48h, to avoid the peak of synaptogenesis in CA1 pyramidal cells that occurs at DIV 7–8 in rat hippocampal slice cultures.^58^

#### Organoid transduction and secretome incubation

At day 46, cortical organoids were transferred to a 1.5 mL centrifuge tube, media carefully aspirated and 200 μL of Neurobasal A (Gibco), supplemented with 2% B27 Plus vitamin A (Gibco), 1% Penicillin-Streptomycin (Gibco), 1% Glutamax (Gibco) and AVV9-hSYN-EGFP (Addgene) at a 1.5 x 10^10^ Vv/mL titter, was added to the tube and incubated at 37°C/5%CO_2_. The following day, 800 μL of virus-free supplemented Neurobasal A was added to the centrifuge tube and returned to the incubator. After 24h the organoid was finally removed from the centrifuge tube and transferred to a 24-well flat bottom Low attachment plate (Grainer) and 2 mL of supplemented Neurobasal A was added. Media was changed every 3 days until day 59 where HUCPVC secretome was diluted in fresh culture media in order to be 2 times concentrated. The organoid was incubated in secretome-rich media for 24h before being harvested.

#### Immunocytochemistry

After stimulation with HUCPVC secretome, cells were fixed in 4% paraformaldehyde (in PBS with 4% sucrose) for 10 min at room temperature, rinsed 3 times for 5 min with PBS and then permeabilized using PBS with 0.25% Triton X-100 for 5 min. Following a brief wash with PBS to remove the permeabilization solution, non-specific binding sites were blocked with 3% Bovine serum albumin (BSA) in PBS for 40 min at room temperature. Afterward, cells were incubated overnight at 4°C with primary antibodies diluted in PBS with 3% of BSA. Primary antibodies were removed by washing the cells 3 times with PBS, and then cells were incubated for 1 h at room temperature with Alexa-conjugated secondary antibodies diluted in PBS with 3% of BSA. Finally, cells were washed twice in PBS with 0.1% Triton X-100 and once in PBS. Coverslips were then mounted in ProLong Gold Antifade mounting medium (Molecular probes, Thermo Fisher Scientific) and preparations were cured overnight at 4°C protected from light and sealed with nail polish.

Organotypic slice cultures were fixed in 4% paraformaldehyde (in PBS with 4% sucrose) during an overnight incubation at 4°C, as previously described.^59^ Afterward, slices were washed 3 times for 10 min with PBS, treated with PBS with 0.1% glycine for 15 min at room temperature, and then permeabilized using PBS with 0.5% Triton X-100 for 3h at room temperature. Non-specific binding sites were blocked with 10% BSA/10% goat serum (GS) in PBS with 0.2% Triton X-100 overnight at 4°C. Then, the slices were incubated overnight at 4°C with primary antibodies diluted in PBS with 5% BSA/5% GS and 0.1% Triton X-100. On the following day, primary antibodies were removed by washing the slices 4 times with PBS containing 0.1% Triton X-100 for 7 min, and then they were incubated for 2 h at room temperature with Alexa-conjugated secondary antibodies diluted in PBS with 5% BSA/5% GS and 0.1% Triton X-100. The secondary antibodies were removed by washing the slices 4 times with PBS containing 0.1% Triton X-100 for 7 min. Finally, the slices were transferred to the slides, mounted in ProLong Gold Antifade mounting medium (Molecular probes, Thermo Fisher Scientific), and covered with the coverslip. Preparations were kept at 4°C until microscopy analysis.

Organoids were fixed in 4% paraformaldehyde for 2 h at room temperature, washed 3 times for 10 min with PBS, and then cryoprotected by immersion in 30% sucrose for 2 days at 4°C. Afterward, organoids were involved in optimal cutting temperature (OCT) compound and stored at −80°C until use. For immunofluorescence staining, 20 μm thick sections were cut using a CryoStar NX50 Cryostat (Thermo Fisher Scientific), collected on SuperFrost Plus slides, and stored at −20°C until use. Cryosections were rinsed 3 times for 5 min with PBS to remove excess OCT, permeabilized with 0.2% Triton X-100 in PBS for 10 min and then blocked with 10% goat serum in PBS with 0.2% Triton X-100 for 1 h at room temperature. The sections were then incubated overnight at 4°C with primary antibodies diluted in PBS containing 0.2% Triton X-100 and 5% goat serum. Primary antibodies were removed by washing the sections 3 times with PBS with 0.1% Triton X-100 for 10 min, and then they were incubated for 2 h at room temperature with Alexa-conjugated secondary antibodies diluted in PBS with 0.2% Triton X-100 and 5% goat serum. Cryosections were then washed 3 times with PBS with 0.1% Triton X-100 for 10 min, mounted with Mountant Permafluor (Thermo Fisher Scientific) and covered with the coverslip. Slides were kept at 4°C until microscopy analysis. The following antibodies and respective dilutions were used: chicken anti-Tau (1:500; Abcam), chicken anti-MAP2 (1:10,000; Abcam), mouse anti-β-Tubulin III (1:1000; Sigma), mouse anti-PSD95 (1:500; Invitrogen, Thermo Fisher Scientific), rabbit anti-Synapsin I (1:4000; Millipore), mouse anti-VGluT1 (1:500; Millipore), guinea pig anti-VGluT1 (1:1500; Millipore), rabbit anti-synaptophysin (1:1000; abcam), chicken anti-GFP (1:1000, abcam), rabbit anti-vimentin (1:200; abcam) and mouse anti-Ki67 (1:100; BD Biosciences). As for secondary antibodies, Alexa Fluor 488-, 568- and 647-conjugated antibodies (1:1000; Thermo Fisher Scientific) were used.

#### Fluorescence microscopy and quantification

Fluorescent images of fixed cells were obtained with a Plan-Apochromat 20x air objective (0.8 numerical aperture) or a Plan-Apochromat 63x oil objective (1.4 numerical aperture) in a Zeiss Observer Z.1 microscope equipped with an AxioCam HRm camera and the Zen Blue 2011 software (ZEISS). Images of random fields of view (FOVs) of either distal axons or the entire neuron were taken under identical settings (exposure time and excitation light intensity were kept constant in each experiment) and converted to 8-bit before any type of analysis. Fluorescent images of banker cultures, hippocampal slices, and organoid sections were obtained with a Plan-Apochromat 63x oil objective (1.4 numerical aperture) in a Carl Zeiss LSM 710 confocal microscope equipped with a QUASAR detection unit and the Zen Black 2012 software (ZEISS). For all experimental conditions, images of banker cultures, organoid sections and images of the *stratum oriens* layer of the hippocampal CA1 region were acquired using the same settings (gain and laser power were kept constant in each independent experiment). Quantifications were performed using ImageJ/Fiji software (National Institutes of Health) or the ICY software^60^ in the case of organotypic slices and organoid sections. 3D reconstructions were performed in the Imaris software (Oxford Instruments).

To quantify the number of axonal branches, the axonal marker was used to measure each ramification that extends from the longest axon segment of each neuron. Ramifications whose length was equal or higher than 5 μm, were considered as axonal branches. The number of branches was divided by the total axonal length.

To determine the number of presynaptic clusters/puncta along the axons, the axonal marker was used to randomly select populations of axons to quantify. First, the axonal length was obtained by analysing a “skeletonized” version of the axonal marker, using the ImageJ plugin “Analyze skeleton”. The sum of the length of all the axonal branches identified within an image was used as the axonal length. Then, corresponding images of synaptic vesicle markers were submitted to a thresholding and a particle analysis was performed to obtain puncta number. The number of puncta was then divided by the calculated axonal length.

To quantify axodendritic synapses (PSD95-Synapsin clusters), z stack images were obtained at 63x magnification (z intervals of 0.2 μm, 7 sections/z stack) and assembled into a single image using a maximum intensity projection. The dendritic marker MAP2 was used to measure each dendrite that extends from the cell body and the sum of the length of all dendrites identified within each image was used as the dendritic length. Then, corresponding images of synaptic markers were submitted to thresholding and a particle analysis was performed to calculate their number and to determine the regions of interests (ROIs) of each punctum. The presence or absence of Synapsin signal within PSD95 puncta ROIs was determined, and the total number of PSD95 ROIs containing Synapsin was divided by the dendritic length to determine the number of clusters (PSD95 puncta that co-localizes with Synapsin puncta) per dendritic length.

To determine the number and intensity of presynaptic clusters in hippocampal slices, z stack images were obtained at 63x magnification (z intervals of 0.45 μm, 7 sections/z stack) and analyzed using the ICY software plugin “Spot Detector”, which extracts spots based on an undecimated wavelet transform detector. The total number and mean intensity of VGluT1 clusters were extracted from each image and normalized for the ROI area. Analyses were done on maximum projections of z-stacks.

To calculate the number of presynaptic sites in organoid sections, z stack images (z intervals of 0.41 μm, 11 sections/z stack in a 20 μm thick section) were obtained with a 63x oil objective and assembled into a single image using a maximum intensity projection. Analyses were carried out on ICY software using the “Spot Detector” plugin, and colocalization of presynaptic markers was conducted based on the statistical method SODA (Statistical Object Distance Analysis) within the same software. The number of synaptophysin puncta containing VGluT1 was normalized for the section area. 2–3 images per section from 3 to 4 cryosections of each organoid, from 3 different organoids per group were analyzed.

All images were processed and prepared for presentation using ImageJ/Fiji, Imaris and Illustrator (Adobe) software.

#### MEA electrophysiology recordings

The neuronal network activity in hippocampal cultures grown on MEAs was sampled at 10 kHz using the MEA2100 *in vitro* recording system (MCS GmbH, Germany). The 6-well MEA containing cultured neurons was placed on the recording system and allowed to stabilize for 5 min before any data acquisition. Then, the baseline neuronal activity was recorded during 30 min. Throughout the entire time, cells were maintained at 37°C under a humidified atmosphere of 5% CO_2_ using external controllers (ibidi GmbH, Germany). Afterward, half of the wells on each MEA were stimulated with HUCPVC secretome and the other half with fresh Neurobasal medium. The MEA was then returned to the incubator for 6 h. After that period, new recordings of 30 min were performed, as described previously. On both cases, recordings were obtained via the Multi-Channel Experimenter software (Multi Channel Systems MCS GmbH, Germany), where the raw signal was high-pass filtered (200 Hz) and low-pass filtered (3500 Hz). Spikes were detected by a threshold set to 5x the standard deviation (SD) of the electrode noise and the spike-time data was saved. The mean firing rate (MFR) of each electrode was calculated in MATLAB 2020a (The MathWorks Inc., USA) using custom scripts. Electrodes with an MFR of at least 0.1 Hz were considered as active and included in the analysis. The activity of each culture was determined by averaging the MFR of total active electrodes.

#### Patch-clamp electrophysiology

The activity of excitatory synapses was evaluated by recording miniature (action potential-independent) excitatory postsynaptic currents (mEPSCs) selectively mediated by AMPA receptors. Glass coverslips containing cultured neurons were mounted on a recording chamber and perfused with extracellular solution containing 140 mM NaCl, 10 mM glucose, 2.4 mM KCl, 4 mM MgCl_2_, 4 mM CaCl_2_ and 10 mM HEPES, with a pH of 7.3 (adjusted with NaOH/HCl) and osmolarity of 300–310 mOsM (adjusted with NaCl/water) at an approximate rate of 2 mL/min. This solution was supplemented with tetrodotoxin (TTX, 500 nM, Tocris), bicuculline (10 μM, Tocris) and D-2-amino-5-phosphonovaleric acid (D-AP5, 50 μM, Tocris) to block voltage-gated sodium channels, GABAA receptors and NMDA receptors, respectively. Neurons were visualized under phase contrast on a Zeiss Axio Observer A1 inverted microscope equipped with a 40x oil immersion objective. Patch pipettes were pulled from borosilicate glass and had a tip resistance of 4–6 MOhm when filled with a caesium-based intracellular solution containing 135 mM CsMeSO_4_, 6 mM NaCl, 10 mM HEPES, 0.25 mM EGTA, 2 mM Na_2_ATP, 2 mM MgCl_2_, 0.3 mM Na_2_GTP and 7 mM phosphocreatine, pH 7.3 (adjusted with CsOH) and osmolarity of 300 mOsM (adjusted with CsMeSO_4_/water). Voltage-clamp recordings were performed from cells clamped at −70 mV using an EPC10 double amplifier controlled by the PatchMaster software (HEKA, Germany). The data was filtered at 2.9 kHz and sampled at 10 kHz. The recordings were analyzed offline in ClampFit to assess the frequency and amplitude of mEPSCs using an automated routine based on template matching (obtained by selection, automatic alignment and averaging of at least 20 visually identified synaptic events). Only cells with a stable series resistance of <20 MOhm were accepted for analysis and no series resistance compensation was performed.

### Quantification and statistical analysis

Results are presented as the mean ± SEM of at least three independent biological replicates (independent cultures or organoids). When applicable, for each independent experiment, data was normalized to the mean of the control group and expressed as % of control. Graphs and statistical analysis were performed in Graph Pad Prism 8 software. All statistical information, including replicates, statistical test and *p* value, is reported in the figure legends. For statistical analysis of two groups, either an unpaired t-test or a Mann-Whitney unpaired test was used, depending on whether the data followed a normal distribution. To analyze the difference of multiple groups, one-way ANOVA followed by Bonferroni’s multiple comparisons test was used. Values of *p* < 0.05 were considered statistically significant.

## References

[1] Geoffroy C.G., Zheng B. (2014). Myelin-associated inhibitors in axonal growth after CNS injury. Curr. Opin. Neurobiol..

[2] Bradbury E.J., Burnside E.R. (2019). Moving beyond the glial scar for spinal cord repair. Nat. Commun..

[3] Tomé D., Almeida R.D. (2024). The injured axon: intrinsic mechanisms driving axonal regeneration. Trends Neurosci..

[4] Nombela-Arrieta C., Ritz J., Silberstein L.E. (2011). The elusive nature and function of mesenchymal stem cells. Nat. Rev. Mol. Cell Biol..

[5] Gögel S., Gubernator M., Minger S.L. (2011). Progress and prospects: stem cells and neurological diseases. Gene Ther..

[6] Maltman D.J., Hardy S.A., Przyborski S.A. (2011). Role of mesenchymal stem cells in neurogenesis and nervous system repair. Neurochem. Int..

[7] Cofano F., Boido M., Monticelli M., Zenga F., Ducati A., Vercelli A., Garbossa D. (2019). Mesenchymal Stem Cells for Spinal Cord Injury: Current Options, Limitations, and Future of Cell Therapy. Int. J. Mol. Sci..

[8] Muñoz-Elias G., Marcus A.J., Coyne T.M., Woodbury D., Black I.B. (2004). Adult Bone Marrow Stromal Cells in the Embryonic Brain: Engraftment, Migration, Differentiation, and Long-Term Survival. J. Neurosci..

[9] Deng J., Petersen B.E., Steindler D.A., Jorgensen M.L., Laywell E.D. (2006). Mesenchymal Stem Cells Spontaneously Express Neural Proteins in Culture and Are Neurogenic after Transplantation. Stem Cell..

[10] Martins L.F., Costa R.O., Pedro J.R., Aguiar P., Serra S.C., Teixeira F.G., Sousa N., Salgado A.J., Almeida R.D. (2017). Mesenchymal stem cells secretome-induced axonal outgrowth is mediated by BDNF. Sci. Rep..

[11] Kolar M.K., Itte V.N., Kingham P.J., Novikov L.N., Wiberg M., Kelk P. (2017). The neurotrophic effects of different human dental mesenchymal stem cells. Sci. Rep..

[12] Assunção-Silva R.C., Mendes-Pinheiro B., Patrício P., Behie L.A., Teixeira F.G., Pinto L., Salgado A.J. (2018). Exploiting the impact of the secretome of MSCs isolated from different tissue sources on neuronal differentiation and axonal growth. Biochimie.

[13] Huang J.-H., Yin X.-M., Xu Y., Xu C.-C., Lin X., Ye F.-B., Cao Y., Lin F.-Y. (2017). Systemic Administration of Exosomes Released from Mesenchymal Stromal Cells Attenuates Apoptosis, Inflammation, and Promotes Angiogenesis after Spinal Cord Injury in Rats. J. Neurotrauma.

[14] Vizoso F.J., Eiro N., Cid S., Schneider J., Perez-Fernandez R. (2017). Mesenchymal Stem Cell Secretome: Toward Cell-Free Therapeutic Strategies in Regenerative Medicine. Int. J. Mol. Sci..

[15] Pires A.O., Mendes-Pinheiro B., Teixeira F.G., Anjo S.I., Ribeiro-Samy S., Gomes E.D., Serra S.C., Silva N.A., Manadas B., Sousa N. (2016). Unveiling the Differences of Secretome of Human Bone Marrow Mesenchymal Stem Cells, Adipose Tissue-Derived Stem Cells, and Human Umbilical Cord Perivascular Cells: A Proteomic Analysis. Stem Cells Dev..

[16] Zhang Y., Chopp M., Liu X.S., Katakowski M., Wang X., Tian X., Wu D., Zhang Z.G. (2017). Exosomes Derived from Mesenchymal Stromal Cells Promote Axonal Growth of Cortical Neurons. Mol. Neurobiol..

[17] Tran C., Damaser M.S. (2015). Stem cells as drug delivery methods: Application of stem cell secretome for regeneration. Adv. Drug Deliv. Rev..

[18] Pinto M.J., Almeida R.D. (2016). Puzzling out presynaptic differentiation. J. Neurochem..

[19] Tomé D., Almeida R.D. (2024). Remaking a connection: molecular players involved in post-injury synapse formation. Neural Regen. Res..

[20] Jacobi A., Loy K., Schmalz A.M., Hellsten M., Umemori H., Kerschensteiner M., Bareyre F.M. (2015). FGF22 signaling regulates synapse formation during post-injury remodeling of the spinal cord. EMBO J..

[21] Tyzack G.E., Sitnikov S., Barson D., Adams-Carr K.L., Lau N.K., Kwok J.C., Zhao C., Franklin R.J.M., Karadottir R.T., Fawcett J.W. (2014). Astrocyte response to motor neuron injury promotes structural synaptic plasticity via STAT3-regulated TSP-1 expression. Nat. Commun..

[22] Mauri M., Lentini D., Gravati M., Foudah D., Biella G., Costa B., Toselli M., Parenti M., Coco S. (2012). Mesenchymal stem cells enhance GABAergic transmission in co-cultured hippocampal neurons. Mol. Cell. Neurosci..

[23] Sarugaser R., Lickorish D., Baksh D., Hosseini M.M., Davies J.E. (2005). Human Umbilical Cord Perivascular (HUCPV) Cells: A Source of Mesenchymal Progenitors. Stem Cell..

[24] Christopherson K.S., Ullian E.M., Stokes C.C.A., Mullowney C.E., Hell J.W., Agah A., Lawler J., Mosher D.F., Bornstein P., Barres B.A. (2005). Thrombospondins are astrocyte-secreted proteins that promote CNS synaptogenesis. Cell..

[25] Eroglu Ç., Allen N.J., Susman M.W., O’Rourke N.A., Park C.Y., Özkan E., Chakraborty C., Mulinyawe S.B., Annis D.S., Huberman A.D. (2009). Gabapentin Receptor α2δ-1 Is a Neuronal Thrombospondin Receptor Responsible for Excitatory CNS Synaptogenesis. Cell.

[26] Risher W.C., Kim N., Koh S., Choi J.-E., Mitev P., Spence E.F., Pilaz L.-J., Wang D., Feng G., Silver D.L. (2018). Thrombospondin receptor α2δ-1 promotes synaptogenesis and spinogenesis via postsynaptic Rac1. J. Cell Biol..

[27] Gibson D.A., Ma L. (2011). Developmental regulation of axon branching in the vertebrate nervous system. Development.

[28] Dominici M., Le Blanc K., Mueller I., Slaper-Cortenbach I., Marini F.C., Krause D.S., Deans R.J., Keating A., Prockop D.J., Horwitz E.M. (2006). Minimal criteria for defining multipotent mesenchymal stromal cells. The International Society for Cellular Therapy position statement. Cytotherapy.

[29] Gogolla N., Galimberti I., DePaola V., Caroni P. (2006). Preparation of organotypic hippocampal slice cultures for long-term live imaging. Nat. Protoc..

[30] Chevaleyre V., Siegelbaum S.A. (2010). Strong CA2 pyramidal neuron synapses define a powerful disynaptic cortico-hippocampal loop. Neuron.

[31] Taylor A.M., Blurton-Jones M., Rhee S.W., Cribbs D.H., Cotman C.W., Jeon N.L. (2005). A microfluidic culture platform for CNS axonal injury, regeneration and transport. Nat. Methods.

[32] Pinto M.J., Pedro J.R., Costa R.O., Almeida R.D. (2016). Visualizing K48 Ubiquitination during Presynaptic Formation By Ubiquitination-Induced Fluorescence Complementation (UiFC). Front. Mol. Neurosci..

[33] Costa R.O., Martins H., Martins L.F., Cwetsch A.W., Mele M., Pedro J.R., Tomé D., Jeon N.L., Cancedda L., Jaffrey S.R. (2019). Synaptogenesis Stimulates a Proteasome-Mediated Ribosome Reduction in Axons. Cell Rep..

[34] Johnson-Venkatesh E.M., Umemori H. (2010). Secreted factors as synaptic organizers. Eur. J. Neurosci..

[35] Sabo S.L., Gomes R.A., McAllister A.K. (2006). Formation of Presynaptic Terminals at Predefined Sites along Axons. J. Neurosci..

[36] Kim D.H., Lim H., Lee D., Choi S.J., Oh W., Yang Y.S., Oh J.S., Hwang H.H., Jeon H.B. (2018). Thrombospondin-1 secreted by human umbilical cord blood-derived mesenchymal stem cells rescues neurons from synaptic dysfunction in Alzheimer’s disease model. Sci. Rep..

[37] Xu J., Xiao N., Xia J. (2010). Thrombospondin 1 accelerates synaptogenesis in hippocampal neurons through neuroligin 1. Nat. Neurosci..

[38] Yu Y.P., Gong N., Kweon T.D., Vo B., Luo Z.D. (2018). Gabapentin prevents synaptogenesis between sensory and spinal cord neurons induced by thrombospondin-4 acting on pre-synaptic Cav α2δ1 subunits and involving T-type Ca2+ channels. Br. J. Pharmacol..

[39] Annis D.S., Murphy-Ullrich J.E., Mosher D.F. (2006). Function-blocking antithrombospondin-1 monoclonal antibodies. J. Thromb. Haemost..

[40] Kalil K., Dent E.W. (2014). Branch management: Mechanisms of axon branching in the developing vertebrate CNS. Nat. Rev. Neurosci..

[41] Tomé D., Dias M.S., Correia J., Almeida R.D. (2023). Fibroblast growth factor signaling in axons: from development to disease. Cell Commun. Signal..

[42] Ratnayaka A., Marra V., Branco T., Staras K. (2011). Extrasynaptic vesicle recycling in mature hippocampal neurons. Nat. Commun..

[43] Kim D.H., Lee D., Chang E.H., Kim J.H., Hwang J.W., Kim J.-Y., Kyung J.W., Kim S.H., Oh J.S., Shim S.M. (2015). GDF-15 Secreted from Human Umbilical Cord Blood Mesenchymal Stem Cells Delivered Through the Cerebrospinal Fluid Promotes Hippocampal Neurogenesis and Synaptic Activity in an Alzheimer’s Disease Model. Stem Cells Dev..

[44] Risher W.C., Eroglu C. (2020). Emerging roles for α2δ subunits in calcium channel function and synaptic connectivity. Curr. Opin. Neurobiol..

[45] Bikbaev A., Ciuraszkiewicz-Wojciech A., Heck J., Klatt O., Freund R., Mitlöhner J., Enrile Lacalle S., Sun M., Repetto D., Frischknecht R. (2020). Auxiliary α2δ1 and α2δ3 Subunits of Calcium Channels Drive Excitatory and Inhibitory Neuronal Network Development. J. Neurosci..

[46] Chen J., Li L., Chen S.R., Chen H., Xie J.D., Sirrieh R.E., MacLean D.M., Zhang Y., Zhou M.H., Jayaraman V. (2018). The α2δ-1-NMDA Receptor Complex Is Critically Involved in Neuropathic Pain Development and Gabapentin Therapeutic Actions. Cell Rep..

[47] Sceniak M.P., Berry C.T., Sabo S.L. (2012). Facilitation of neocortical presynaptic terminal development by NMDA receptor activation. Neural Dev..

[48] Diniz L.P., Almeida J.C., Tortelli V., Vargas Lopes C., Setti-Perdigão P., Stipursky J., Kahn S.A., Romão L.F., De Miranda J., Alves-Leon S.V. (2012). Astrocyte-induced synaptogenesis is mediated by transforming growth factor β signaling through modulation of d-serine levels in cerebral cortex neurons. J. Biol. Chem..

[49] Geoffroy C.G., Lorenzana A.O., Kwan J.P., Lin K., Ghassemi O., Ma A., Xu N., Creger D., Liu K., He Z. (2015). Effects of PTEN and Nogo Codeletion on Corticospinal Axon Sprouting and Regeneration in Mice. J. Neurosci..

[50] Wang Z., Reynolds A., Kirry A., Nienhaus C., Blackmore M.G. (2015). Overexpression of Sox11 Promotes Corticospinal Tract Regeneration after Spinal Injury While Interfering with Functional Recovery. J. Neurosci..

[51] Liauw J., Hoang S., Choi M., Eroglu C., Choi M., Sun G.H., Percy M., Wildman-Tobriner B., Bliss T., Guzman R.G. (2008). Thrombospondins 1 and 2 are necessary for synaptic plasticity and functional recovery after stroke. J. Cereb. Blood Flow Metab..

[52] Lutz A.K., Pfaender S., Incearap B., Ioannidis V., Ottonelli I., Föhr K.J., Cammerer J., Zoller M., Higelin J., Giona F. (2020). Autism-associated SHANK3 mutations impair maturation of neuromuscular junctions and striated muscles. Sci. Transl. Med..

[53] Cristovão G., Pinto M.J., Cunha R.A., Almeida R.D., Gomes C.A. (2014). Activation of microglia bolsters synapse formation. Front. Cell. Neurosci..

[54] Pinto M.J., Alves P.L., Martins L., Pedro J.R., Ryu H.R., Jeon N.L., Taylor A.M., Almeida R.D. (2016). The proteasome controls presynaptic differentiation through modulation of an on-site pool of polyubiquitinated conjugates. J. Cell Biol..

[55] Kaech S., Banker G. (2006). Culturing hippocampal neurons. Nat. Protoc..

[56] Miura Y., Li M.-Y., Birey F., Ikeda K., Revah O., Thete M.V., Park J.-Y., Puno A., Lee S.H., Porteus M.H. (2020). Generation of human striatal organoids and cortico-striatal assembloids from human pluripotent stem cells. Nat. Biotechnol..

[57] Fletcher T.L., De Camilli P., Banker G. (1994). Synaptogenesis in hippocampal cultures: Evidence indicating that axons and dendrites become competent to form synapses at different stages of neuronal development. J. Neurosci..

[58] Gambrill A.C., Barria A. (2011). NMDA receptor subunit composition controls synaptogenesis and synapse stabilization. Proc. Natl. Acad. Sci. USA.

[59] Salamian A., Legutko D., Nowicka K., Badyra B., Kaźmierska-Grębowska P., Caban B., Kowalczyk T., Kaczmarek L., Beroun A. (2021). Inhibition of Matrix Metalloproteinase 9 Activity Promotes Synaptogenesis in the Hippocampus. Cereb. Cortex.

[60] De Chaumont F., Dallongeville S., Chenouard N., Hervé N., Pop S., Provoost T., Meas-Yedid V., Pankajakshan P., Lecomte T., Le Montagner Y. (2012). Icy: An open bioimage informatics platform for extended reproducible research. Nat. Methods.

