# Supplementary material for: Mesenchymal stromal cell secretome-induced synaptogenesis is mediated by thrombospondin-1
Source: iScience. 2025 Aug 19;28(9):113401. doi: 10.1016/j.isci.2025.113401 (PMC12441700; doi:10.1016/j.isci.2025.113401)
Supplement: Document S1. Figures S1–S3 [file mmc1.pdf]

iScience, Volume *28*

Supplemental information

Mesenchymal stromal cell secretome-induced synaptogenesis is mediated by thrombospondin-1

Diogo Tomé, Luís F. Martins, Miguel Aroso, Henrique Santos, Rui O. Costa, João L. Afonso, Sofia C. Serra, Paulo Aguiar, Carlos B. Duarte, João Peça, Paulo S. Pinheiro, António J. Salgado, and Ramiro D. Almeida

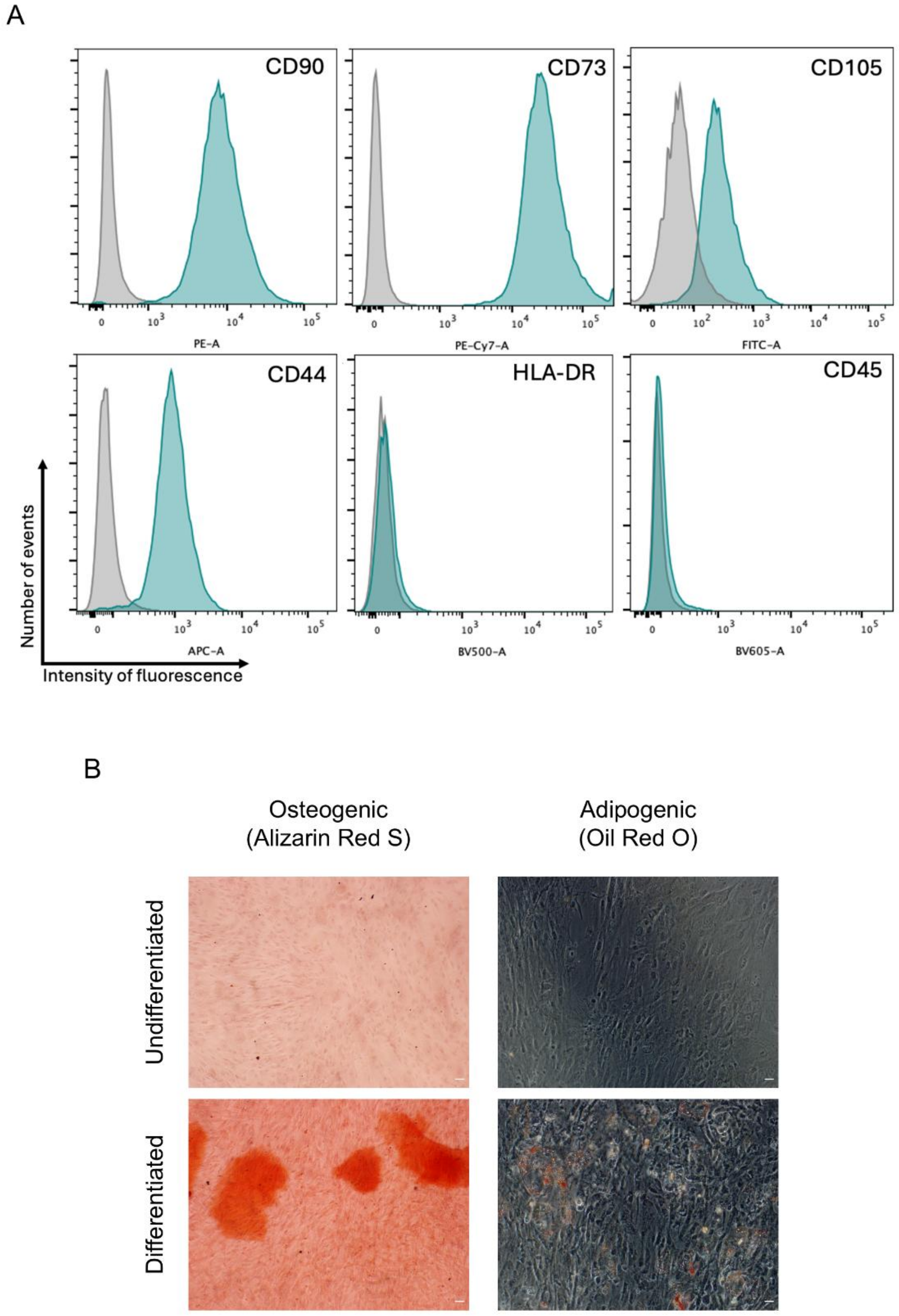

Figure S1. HUCPVC phenotypic and functional characterization

(A) Representative histograms of flow cytometry analysis of HUCPVC (blue) compared to unstained control (gray). Results show that HUCPVC are positive for CD90, CD73, CD105, and CD44 and lack the expression of HLA-DR and CD45 surface markers. Three samples from each group were analysed to plot flow cytometry histograms.

(B) Functional characterization of HUCPVC, depicting their *in vitro* differentiation into osteocytes and adipocytes based on the positive staining for Alizarin Red S (bottom panel) and Oil Red O (bottom panel), respectively. The scale bar is 100 μm.

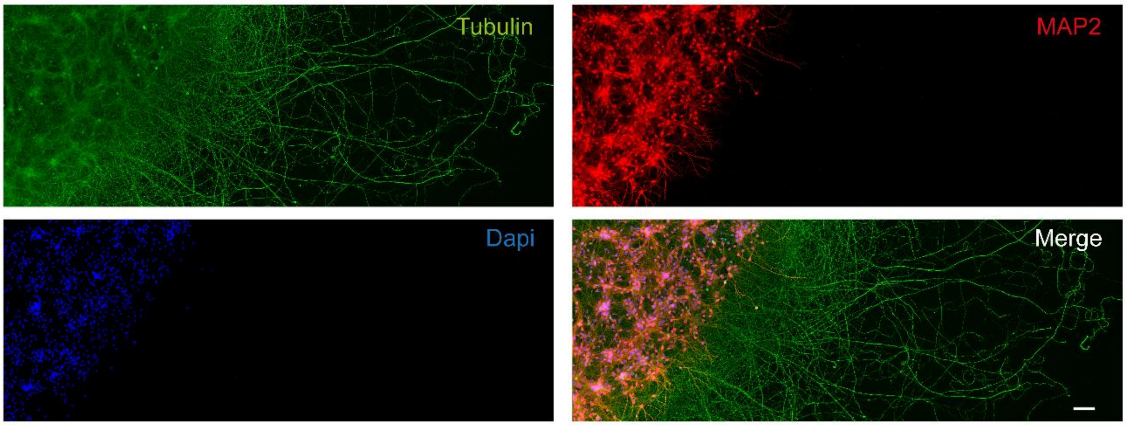

Figure S2. Representative image of a “pseudo-explant” culture, related to figure 2

Hippocampal neurons were immunostained at DIV7 for the neuronal marker βIII-tubulin (green) and the somatodendritic marker MAP2 (red). Nuclei were stained with Dapi (blue). The figure illustrates that this culture system allows the isolation and visualization of distal axons, enabling the study of axon-related mechanisms such as presynaptic differentiation. Contiguous images were taken from an area of the coverslip using an AxioObserver Z1 fluorescent microscope with a PlanApochromat 20x objective and assembled into a single image using ZEN 2011 software. The scale bar is 100 μm.

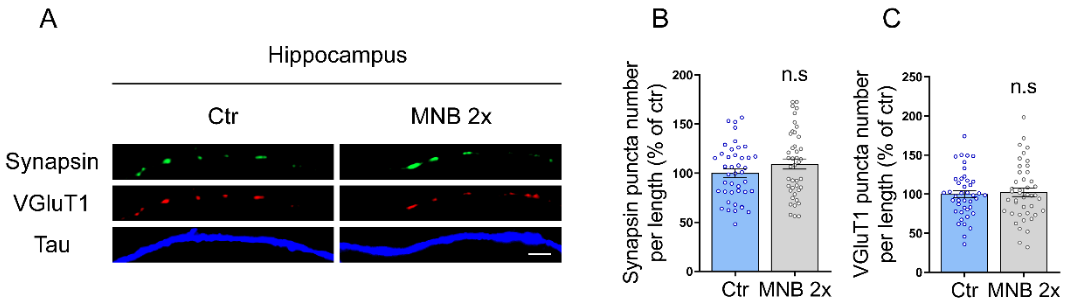

Figure S3. MNB 2X has no effect in presynaptic differentiation of hippocampal neurons, related to figure 2

(A) Effect of MNB 2x concentrated in presynaptic assembly. At DIV7 neurons were stimulated for 6 hours with MNB 2x. The formation of presynaptic clusters was assessed by immunocytochemistry using an antibody against the synaptic vesicle markers synapsin (green) or VGluT1 (red), axons were identified using an antibody against tau (blue). Images were acquired from random axons using an AxioObserver Z1 fluorescent microscope with a PlanApochromat 63x oil objective. The scale bar is 2.5 μm.

(B, C) Quantification of synapsin (B) and VGluT1 (C) puncta number per axonal length. Results demonstrate that global application of MNB 2x for 6 hours has no significant effect in the number of synapsin or VGluT1 puncta, indicating that MNB 2x stimulation does not promote the formation of new presynaptic sites in hippocampal neurons. Puncta analysis was performed with Image J 1.45e software. Results are expressed as % of control. Bars represent the mean ± SEM of 42 images from randomly selected areas of 3 independent experiments; (B, C) **n.s** represents "not significant" by unpaired t-test when compared to Ctr.
